# Recovery of *Vibrio cholerae* polarized cellular organization after exit from a non-proliferating spheroplast state

**DOI:** 10.1371/journal.pone.0293276

**Published:** 2023-10-26

**Authors:** Anthony Goudin, Jean-Luc Ferat, Christophe Possoz, François-Xavier Barre, Elisa Galli

**Affiliations:** Institute for Integrative Biology of the Cell (I2BC), Université Paris-Saclay, CEA, CNRS, Gif-sur-Yvette, France; Centre National de la Recherche Scientifique, Aix-Marseille Université, FRANCE

## Abstract

*Vibrio cholerae*, the causative agent of cholera epidemics, is a rod-shaped bacterium with a highly polarized cellular organization. It can survive harmful growth conditions by entering a non-proliferating spheroplast state, which involves loss of the cell envelope and polarity. How polarized rod organization cells are formed when the spheroplasts exit the non-proliferating state remains largely uncharacterized. To address this question, we investigated how L-arabinose-induced *V*. *cholerae* spheroplasts return to growth. We found that *de novo* morphogenesis started with the elimination of an excess of periplasm, which was immediately followed by cell elongation and the formation of cell branches with a diameter similar to that of normal *V*. *cholerae* cells. Periplasm elimination was driven by bifunctional peptidoglycan synthases involved in cell-wall maintenance, the aPBPs. Elongation and branching relied on the MreB-associated monofunctional peptidoglycan synthase PBP2. The cell division monofunctional peptidoglycan synthase FtsI was not involved in any of these processes. However, the FtsK cell division protein specifically targeted the sites of vesicle extrusion. Genetic material was amplified by synchronous waves of DNA replication as periplasmic elimination began. The HubP polarity factor targeted the tip of the branches as they began to form. However, HubP-mediated polarization was not involved in the efficiency of the recovery process. Finally, our results suggest that the positioning of HubP and the activities of the replication terminus organizer of the two *V*. *cholerae* chromosomes, MatP, are independent of cell division. Taken together, these results confirm the interest of L-arabinose-induced *V*. *cholerae* spheroplasts to study how cell shape is generated and shed light on the *de novo* establishment of the intracellular organization and cell polarization in *V*. *cholerae*.

## Introduction

Prokaryotes have long been perceived as tiny bags of randomly distributed proteins and nucleic acids. However, high-resolution molecular biology and microscopy techniques revealed an extraordinary diversity of cell shapes and developmental programmes, suggesting that bacteria precisely control their form to adapt to specialized ecological niches and/or environmental changes, most notably for pathogenesis [1, 2]. They further revealed that bacterial cells are highly organized and that formation of subcellular domains is critical for numerous cellular processes, including cell division, chromosome segregation and motility [3–8].

The shape of bacteria is maintained by a rigid cell-wall consisting of cross-linked peptidoglycan (PG) [9]. Evolutionary considerations suggest that the Last Bacterial Common Ancestor was probably rod-shaped [10]. In rod-shaped bacteria, the two major bifunctional PG synthases, PBP1a and PBP1b, and the two complexes of monofunctional PG synthases, PBP2/RodA and FtsI/FtsW, mediate PG polymerization and insertion [11]. PBP1a and PBP1b are involved in cell-wall repair and reinforcement [11, 12]. They belong to class A PBPs, referred to as aPBPs. The PBP2/RodA complex is responsible for cell elongation [11]. Its activity is controlled by an actin-like protein, MreB, which moves circumferentially around the cell [13–15]. Together RodAZ, MreBCD and PBP2 form the Rod-complex [11]. The aPBPs together with the Rod-complex form the PG elongation machinery. The FtsI/FtsW complex is part of the cell division apparatus, the divisome, at the core of which lies a ring-like structure, the Z-ring, formed by the tubulin-like protein FtsZ [11, 16–18]. In proteobacteria such as *Escherichia coli* and *Vibrio cholerae*, rod-shaped cells are intrinsically polarized with a ‘new’ pole formed by the division of the parental cell from which they originate, and an ‘old’ pole inherited from one of the two poles of the parental cell [19, 20]. The old pole-new pole polarity serves as a cue for the formation of subcellular domains and for the arrangement of the genetic material [7–11]. In turn, the genetic material is used as a scaffold to position DNA-binding proteins that inhibit the assembly of the Z-ring, which restricts cell division to midcell, where a low DNA density zone is formed between sister chromosomes at the end of the replication/segregation cycle [16, 21–24]. Thus, the shape and intracellular organization of rod-shaped bacteria are maintained in a cell cycle-dependent manner. However, many rod-shaped bacteria transition to non-proliferating cell-wall deficient spherical cells when exposed to environmental insults, such as cell-wall targeting antibiotics, and it remains largely unknown how the shape and intracellular organization are restored when they revert back to proliferation [25–27].

To address this question, we exploited our recent findings that *V*. *cholerae*, the causative agent of the deadly human disease of the same name, forms viable non-proliferating spheroplasts in the presence of L-arabinose (L-Ara), which return to growth within a few hours after L-Ara removal [28]. *V*. *cholerae* belongs to the Vibrionales, whose genome is divided into a primary chromosome, Chr1, and a secondary chromosome, Chr2. The Vibrionales are closely related to the Enterobacterales, such as *E*. *coli*, which are known to be mono-chromosomal bacteria [29]. The primary chromosome of the Vibrionales is derived from the chromosome of the ancestor of the Vibrionales and the Enterobacterales. The secondary chromosome is derived from a megaplasmid. Like most bacterial chromosomes, *V*. *cholerae* Chr1 and Chr2 are circular and carry a single origin of bidirectional replication, *oriC1* and *oriC2*, respectively. Their replication terminates in a zone opposite of their replication origins, *ter1* and *ter2*, respectively. Chr1 harbours a chromosomal partitioning system, ParAB1/*parS1*, which anchors *oriC1* to the old pole of newborn cells and addresses one of the two newly replicated *oriC1* sisters towards the opposite pole during replication. The positioning of *oriC1* depends on a polar transmembrane protein, HubP, which creates a platform for the recruitment of ParA1 and chemotactic and motility factors [30, 31]. Chr2 harbours a plasmid-type partitioning system, ParAB2/*parS2*, which directs *oriC2* to midcell in newborn cells and addresses newly-replicated *oriC2* sisters to quarter positions during replication [32–34]. Chr1 encodes for homologs of most of the proteins implicated in PG synthesis, elongation and cell division in *E*. *coli*, including PBP1a, PBP2, MreB, FtsI and FtsZ [16, 24]. It also encodes for homologs of two site-specific recombinases, XerC and XerD, which add crossovers at a specific site within the terminus of Chr1 and Chr2, *dif1* and *dif2*, respectively, to resolve topological problems due to their circularity such as chromosome dimers [35]. The action of XerC and XerD is under the control of a DNA pump anchored to the divisome, FtsK [33, 35, 36]. Finally, Chr1 encodes for a homolog of MatP, the *E*. *coli* terminus organization protein, which maintains sister *dif1* sites at midcell and sister *dif2* sites close to midcell [33].

Here, we used live fluorescence video-microscopy to follow the morphological changes and choreography of different intracellular machineries during the reversion of L-Ara-induced *V*. *cholerae* spheroplasts to proliferating rod-shaped cells.

## Results

### Chronology of morphological changes

**Fig 1:**
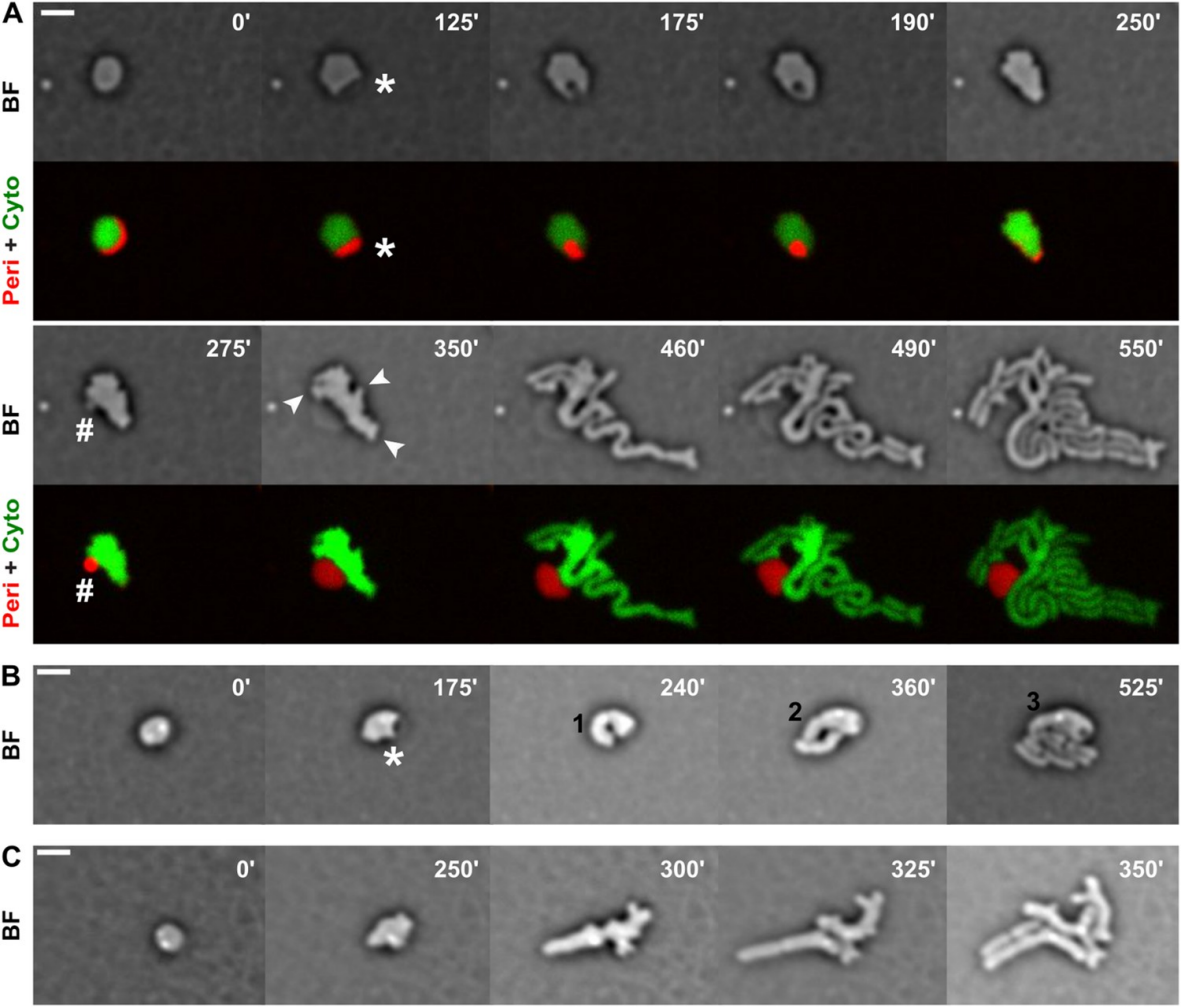
Mechanisms of cell shape recovery. Reconstructed time-lapse bright-field (BF) images of *V*. *cholerae* N16961 derivatives cells grown at 30°C on M9-MM agarose pads after L-Ara removal. One frame was taken every 5 minutes. On the top-right corner of each frame is indicated the time in minutes. Scale bars = 2 μm. **AB.** Standard mechanism of cell shape recovery with periplasmic engulfment. The star points to the periplasmic excess, the hash to the periplasmic vesicle and arrows to cell branches. In cells failing to complete periplasmic engulfment (B), rod-shaped cells are formed at the opposite side of the periplasmic excess and separated by the original cells by cytokinetic events. The numbers in black indicate the number of cells originated in such a way during the recovery process. (A) Strain EGV616: periplasm (Peri) tagged in red and cytoplasm (Cyto) in green, respectively. **C.** Alternative mechanism of cell shape recovery. Spherical cells return to rod shape by an elongation and subsequent division event, without any apparent periplasmic engulfment event.

Out of a total of 820 reversion events observed on M9-minimal medium agarose pads, 93.5% began with the formation of a translucent bulge on one side of the cell (Fig 1A, white star in panel 125’, and 1B, white star in panel 175’). In the vast majority of cases, the bulge appeared to be engulfed and subsequently expelled as a translucent vesicle (Fig 1A, white hash in panel 275’, S1–S3 Movies). Engulfment failed to be completed (Fig 1B, S4 and S5 Movies) or was only achieved after several attempts (S6 Movie) in 6% of the cases. To further characterize the engulfment step, we engineered a strain in which the cytoplasm was labelled by the production of a green fluorescent protein (YGFP) and the periplasm by the production of a red fluorescent protein (mCherry) fused to a membrane export signal. We observed that the transition process from rod to sphere after L-Ara addition began with the formation of a single bleb on the surface of the rod cell with an enlarged crescent moon-shaped periplasmic space (S7 Movie). The periplasmic excess increased in size until the entire cellular content escaped the cell envelope and was assimilated into the spheroplast (S7 Movie). Visualization of the recovery process demonstrated that translucent bulges originated from the pre-existing crescent-shaped periplasmic space of spheroplasts, which was engulfed and subsequently expelled from the cell in the form of vesicles (Fig 1A, white hash in the 275’ panel, S1 Movie). Cells with a wild-type curved rod morphology were only recovered after multiple elongation and division cycles. In cells that completed periplasmic engulfment, early cytokinetic events often produced cells with an abnormal shape. In addition, cytoplasmic bulges formed at the surface of the cells (Fig 1A, white arrows, 350’ panel). They elongated to form branches with a diameter equivalent to that of normal *V*. *cholerae* cells, which divided into rod-shaped offspring. The timing and frequency of vesicle extrusion, branch formation and division events varied in the different cells (S1–S3 Movies). Formation of cytoplasmic bulges elongating in cell branches were also characteristic of the sphere to rod transition described for *E*. *coli* [37] and *Bacillus subtilis* [38] cells. In cells that did not complete periplasmic engulfment, a curved cylindrical protrusion appeared juxtaposed to the site of periplasmic excess and rapidly elongated into a nascent rod, with cytokinesis directly giving rise to new rod-shaped bacteria (Fig 1B, S4–S6 movies).

In the remaining 6.5% of the reversion events, no periplasmic excess was detected. Early divisions originated thick elongated cells with aberrant morphologies and protruding branches. Successive elongation and division steps eventually led to the formation of rod-shaped bacteria (Fig 1C, S8–S10 Movies).

### Morphological changes are linked to new PG synthesis and insertion

We inspected the recovery dynamics of spheroplasts in conditions that selectively inhibited each of the different cell-wall synthetic machineries to determine their relative importance for the formation of rod-shaped proliferating cells.

**Fig 2:**
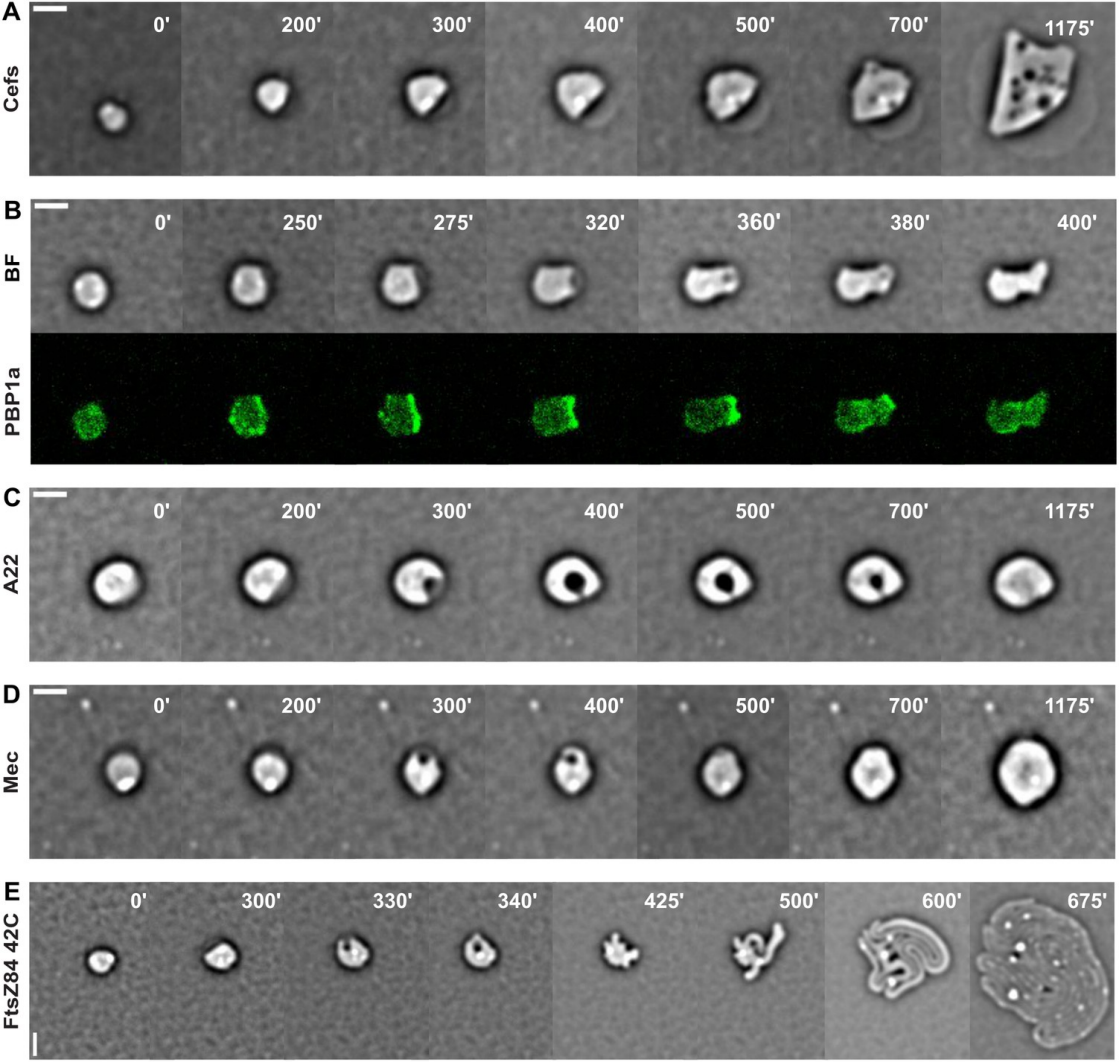
Role of cell-wall synthesis in cell shape recovery. Reconstructed time-lapse BF and fluorescent images. N16961 derivative cells were grown on M9-MM agarose pads at 30°C (ABCD) or 42°C (E) after L-Ara removal. One frame was taken every 5 minutes. On the top-right corner of each frame is indicated the time in minutes. Scale bars = 2 μm. **ACD.** Recovery of cells in presence of 1 mg/ml cefsulodin (Cefs) (A), 10 μg/ml A22 (C), 10 μg/ml mecillinam (Mec) (D). **B.** Localization of PBP1a-sfGFP. **E.** Recovery of rod shape of cells carrying the *ftsZ84* temperature sensitive mutation.

First, we used cefsulodin, a β-lactam antibiotic that arrests the proliferation of *V*. *cholerae* cells by specifically inhibiting bifunctional PBPs [39]. Treatment of proliferating rod-shaped *V*. *cholerae* cells with cefsulodin led to the formation of a bleb on the cell surface at random positions. The bleb expanded in size until all the cell material escaped the cell envelope (S11 Movie). Treatment of L-Ara-induced spheroplasts with cefsulodin impeded periplasmic engulfment and sphere elongation during recovery (Fig 2A and S12 Movie). The spheroplasts only increased in diameter (Fig 2A and S12 Movie). The major bifunctional PBP of *V*. *cholerae* is PBP1a [40, 41]. We engineered a functional C-terminal fusion of PBP1a with sfGFP to follow its localization during the *de novo* formation of rod-shaped cells. PBP1a-sfGFP was diffused in L-Ara-induced spheroplasts (Fig 2B, 0’ panel). At the onset of the recovery process, bright patches and spots appeared around the circumference of the spheroplasts (Fig 2B, 250’ panel). They concentrated on the side of the translucent crescent-shaped periplasmic space of the spheroplasts and remained at its leading edge during the engulfment and elimination process (Fig 2B and S13 Movie).

Second, we specifically arrested the action of the Rod-complex with A22, which inhibits the activity of MreB [42], or mecillinam, which inactivates PBP2 [43]. Treating proliferating rod-shaped *V*. *cholerae* cells with A22 or mecillinam blocked cell elongation and led to the formation of characteristic lemon-shaped cells that became spherical and increased in size until lysis (S14 and S15 Movies, respectively). Treating L-Ara-induced spheroplasts with A22 or mecillinam did not impede growth restart. However, periplasmic engulfment took a longer time (Fig 2C and 2D, respectively). In addition, inhibition of the activity of the Rod-complex diminished the increase in volume of the spheroplasts (S16 and S17 Movies). No cell elongation or division event occurred (S16 and S17 Movies).

Finally, we used a temperature sensitive allele of *ftsZ*, *ftsZ84*, to inhibit divisome formation. At the non-permissive temperature (42°C), rod-shaped *V*. *cholerae ftsZ84* cells form long aseptate filaments [16, 44]. At the non-permissive temperature, recovering L-Ara-induced *ftsZ84* spheroplasts performed periplasmic engulfment, elongated and created multiple branches (Fig 2E and S18 Movie). The original spherical cell and the newly formed branches elongated as a unique tentacular cell, which eventually lysed. Before lysis, all connected cell filaments had recovered a cell diameter similar to that of wild-type proliferating cells.

**Fig 3:**
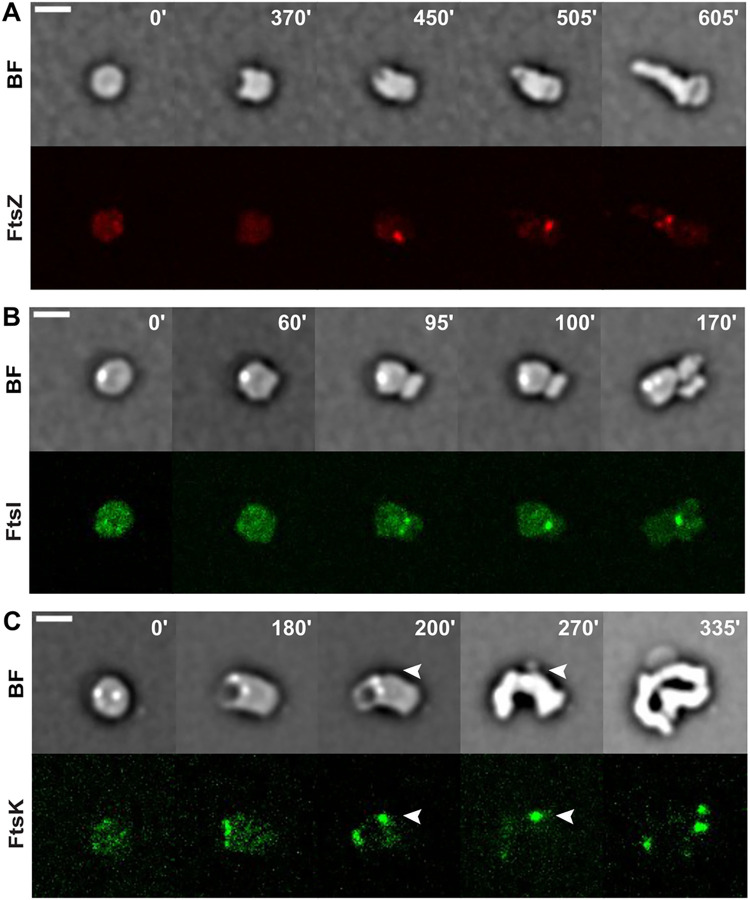
Role of divisome components in rod shape recovery. Reconstructed time-lapse BF and fluorescent images. N16961 derivative cells were grown on M9-MM agarose pads at 30°C after L-Ara removal. One frame was taken every 5 minutes. On the top-right corner of each frame is indicated the time in minutes. Scale bars = 2 μm. **A.** Localization of FtsZ-RFPT. **B.** Localization of YGFP-FtsI. **C.** Localization of FtsK-YGFP.

To further prove that the divisome played an accessory role in the morphological changes, we inspected the localization pattern and dynamics of FtsZ [45], FtsI [46], and FtsK [47, 48] using a partially functional FtsZ-RFPT fusion produced from an ectopic chromosomal locus in presence of the untagged wild-type copy [24] and fully functional YGFP-FtsI and FtsK-YGFP fusions [16]. In L-Ara-induced spheroplasts, FtsZ-RFPT, YGFP-FtsI and FtsK-YGFP were diffused. During recovery, they all localized at the sites of septal constriction (Fig 3, S19–S21 Movies). However, FtsK-YGFP localized also at the site of periplasmic engulfment and encircled the periplasmic excess until its elimination. Furthermore, FtsK-YGFP accumulated at the sites of extrusion of new periplasmic vesicles that were formed after the periplasmic engulfment (Fig 3C white arrows and S21 Movie).

Taken together, these results corroborate the observations made in *V*. *cholerae* cell-wall deficient cells recovering rod shape after treatment with cell-wall targeting antibiotics [49]. In both cases, inhibiting aPBPs prevented the periplasmic engulfment process and successive cell shape recovery steps whereas inhibiting the Rod-complex precluded cell elongation and branching.

In addition, localization of FtsK at the leading edge of periplasmic engulfment and at sites of extrusion of periplasmic vesicles suggests it might play a role at sites where membrane fusion is required. Even though, at present, we cannot exclude FtsK is recruited there by another protein and does not actively participate in the periplasmic engulfment process and in vesicle extrusion.

### Morphological changes are accompanied by waves of replication

In M9-minimal media, *V*. *cholerae* cells contain between 1 and 2 sets of chromosomes. This is reflected in the DNA content of cells measured by flow cytometry (S1A Fig) and the V-shaped profile of the number of copies of each locus as a function of its distance from the origin (MF analysis, S1B Fig). After a 5h incubation with L-Ara, when rods started transitioning to spheroplasts [28], the DNA content of most cells was equivalent to 2 genome copies (S1A Fig, T5). The flow cytometry profile was largely unchanged after an additional incubation period of 2h, i.e. at a time when the majority of cells had transitioned to spheres [28], suggesting replication arrest (S1A Fig, T7). The flat MF profile obtained after 10h of L-Ara treatment confirmed that the two *V*. *cholerae* chromosomes, Chr1 and Chr2, were fully replicated in spherical cells. To confirm the number of chromosome copies per cell, we visualized *oriC1* and *oriC2* loci using two compatible fluorescent reporter systems. To this end, we inserted a *parS*^*pMT1*^ site in proximity of *oriC1* and a *lacO* array in proximity of *oriC2*, which are bound by YGFP-ParB^pMT1^ and LacI-RFPT, respectively. Together with the MF analysis data, it demonstrated that each cell contained an equal number of Chr1 and Chr2 copies (S1C Fig). Thus, in the presence of L-Ara, *V*. *cholerae* cells are able to initiate and terminate a single round of replication, leading to the formation of a majority of spheroplasts with two full copies of Chr1 and Chr2.

During the reversion process, we did not observe any noticeable growth defect such as growth arrest or lysis in the rod-shaped cells first originated from the spheroplasts by elongation or branching. However, we cannot exclude the presence of minor defects not detectable by visual inspection of cell recovery on agarose pads. This suggests the DNA replication and segregation machineries are active and functional since the first stages of the shape recovery process.

**Fig 4:**
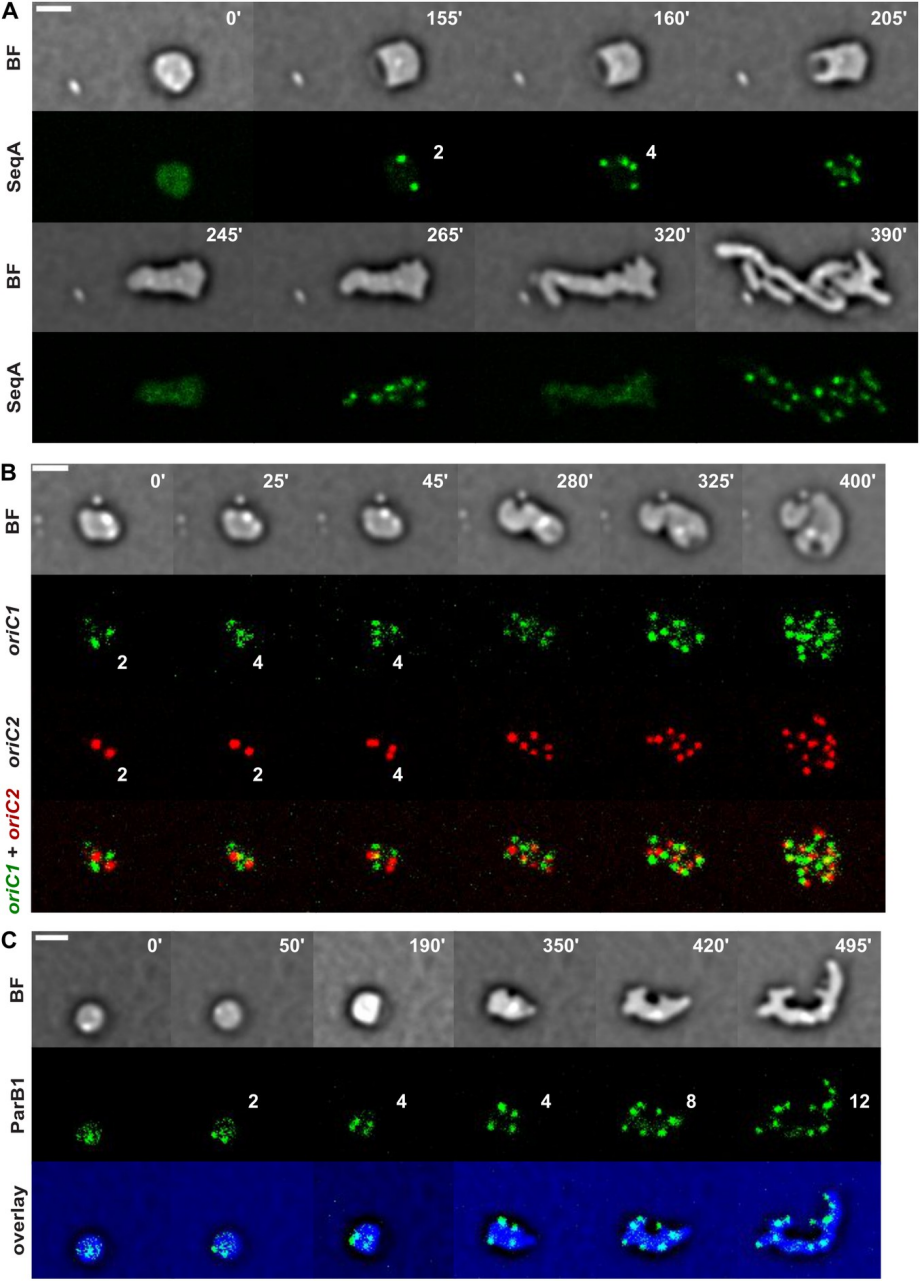
Chromosome replication re-start at the beginning of cell shape recovery. Reconstructed time-lapse BF and fluorescent images. N16961 derivative cells were grown on M9-MM agarose pads at 30°C. One frame was taken every 5 minutes. On the top-right corner of each frame is indicated the time in minutes. Scale bars = 2 μm. The number of SeqA, *oriC1* and *oriC2*, and ParB1 foci is indicated in the time-lapse panels. **A.** Localization of SeqA-YGFP. **B.** *oriC1* and *oriC2* choreographies during cell shape recovery. **C.** Localization of YGFP-ParB1.

To follow the DNA replication process during cell shape recovery and return to proliferation, we constructed a functional SeqA-YGFP fluorescent fusion and inserted it at its chromosomal locus under its native promoter. SeqA is a negative regulator of the initiation of chromosome replication, it binds to newly synthesized hemimethylated DNA behind the replication forks and dissociates after its full methylation, thus reporting on the DNA replication progression and status [50, 51]. At the start of the recovery process, SeqA-YGFP was diffused, confirming the absence of on-going DNA replication (panel 0’ of Fig 4A and S22 Movie). Two SeqA foci appeared soon after L-Ara removal and 2 new foci appeared before the engulfment of the periplasmic excess (Fig 4A, panel 155’ and 160’, respectively). As Chr2 replication initiation is delayed until a locus located in the middle of the left arm of Chr1 is duplicated, it is likely that the 2 SeqA foci that first appeared corresponded to the initiation of replication of the 2 Chr1 copies contained by the spheroplasts and that the 2 SeqA foci that appeared soon after corresponded to the initiation of replication of the 2 Chr2 copies. Correspondingly, *oriC1* foci duplicated first, increasing from 2 to 4 foci (panel 25’ of Fig 4B and S23 Movie), followed by duplication of *oriC2* foci (panel 45’ of Fig 4B and S23 Movie). Furthermore, soon after the appearance of SeqA foci, the fluorescent intensity of the spots decreased and they appeared to split in two adjacent foci, suggesting the presence of a replisome machinery on each arm of Chr1 and Chr2 (S22 Movie). Throughout the recovery process, SeqA foci appeared and disappeared in waves, doubling in number at each time (panels 205’, 245’, 265’ and 320’ of Fig 4A and S22 Movie). Correspondingly, the number of *oriC1* and *oriC2* foci kept doubling during the recovery process. Taken together, these results indicate that DNA replication restarted very early during the sphere to rod reversion and that the coordinated replication programme of Chr1 and Chr2 was maintained [50, 52]. The wave-like pattern of appearance and disappearance of SeqA foci in the elongating spheroplasts further indicated that the new rounds of replication of the different Chr1 an Chr2 sets harboured by each cell were synchronized before cell individualization by division events.

### Sister chromatid individualization is independent of cell shape

The genetic material must not only be replicated but also correctly segregated to ensure that each new cell receives a complete set of chromosomes. Under normal growth conditions, sister copies of the origin regions of Chr1 and Chr2 are segregated to opposite side of the cells by the ParAB1 and ParAB2 partition systems, respectively [16, 32]. As ParB1 binds to specific sites located next to *oriC1*, we could use the position of a ParB1-YGFP fluorescent fusion protein expressed from the leakiness of a P_*lac*_ promoter at an ectopic position as a proxy of the position of *oriC1* sister copies throughout the recovery process (Fig 4C and S24 Movie). In the first stage of the reversion process, when the cell retains a spherical or ovoid shape, ParB1 foci were very mobile and newly duplicated foci randomly moved to different positions within the cell, suggesting that Chr1 copies were individualized. This result was corroborated by the direct observation of the dynamics of *oriC1* foci labelled with the *parS*^*pMT1*^/ParB^pMT1^-YGFP system. Direct observation of the dynamics of *oriC2* foci labelled with the *lacO*/LacI-mCherry system suggested that Chr2 copies were also individualized (Fig 4B and S23 Movie). In the later stages of the recovery process, ParB1 foci localized to the pole of elongating branches and were often present at the position of newly-visible protruding bulges on the cell surface, suggesting that the partition systems could correctly segregate sister chromosomes in the protruding branches (Fig 4C, panels 350’ to 495’, and S24 Movie).

### MatP plays a role in the management of the *ter* domain during cell shape recovery

Flat MF profiles of L-Ara-treated cells indicated that both Chr1 and Chr2 were fully replicated, with no region of the chromosomes in excess compared to others (S1B Fig). However, the simultaneous visualization of *oriC1* and *ter1* loci by binding of YGFP-ParB^pMT1^ to a *parS*^*pMT1*^ site located next to *oriC1* and LacI-RFPT to a *lacO* array next to *ter1* showed that 75% and 25% of the L-Ara-induced spheroplasts with 2 *oriC1* foci had a single *ter1* focus and 2 *ter1* foci, respectively (S1D Fig). The fraction of spheroplasts with 2 *oriC1* foci that contained a single *ter1* focus decreased to 35% whereas those containing 2 *ter1* foci increased to 65% when *matP* was deleted (S1D Fig).

**Fig 5:**
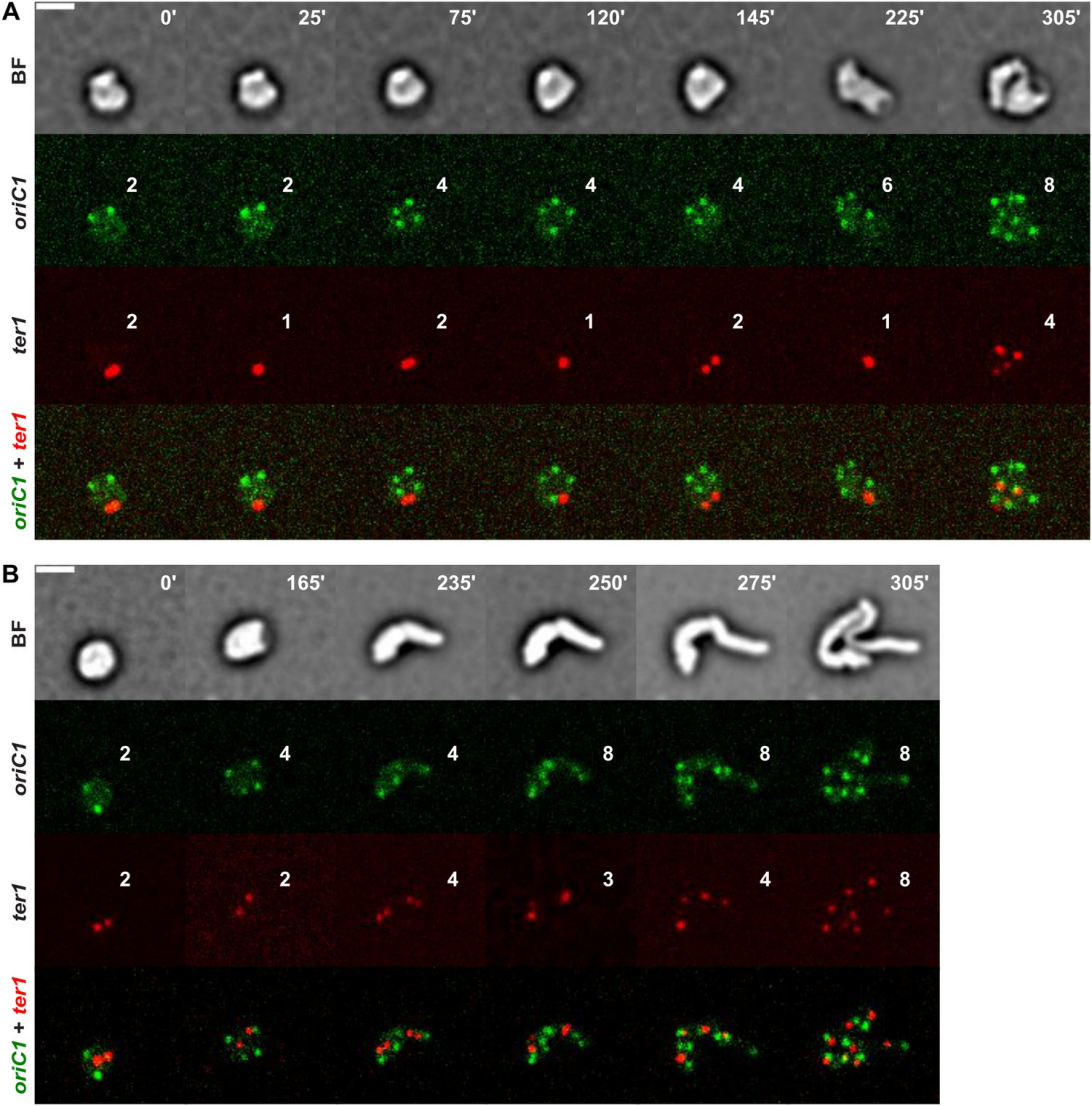
MatP plays a role in *ter1* delayed segregation during cell shape recovery. Reconstructed time-lapse BF and fluorescent images. N16961 derivative cells were grown on M9-MM agarose pads at 30°C. One frame was taken every 5 minutes. On the top-right corner of each frame is indicated the time in minutes. Scale bars = 2 μm. **AB.** Choreographies of *oriC1* and *ter1* in wild-type (A) and Δ*matP* (B) cells. The number of *oriC1* and *ter1* foci is indicated in each panel.

During recovery, newly duplicated *ter1* foci kept getting apart and colliding back together whereas *oriC1* foci moved apart from each other towards opposite cell halves (Fig 5A and S25 Movie). The *oriC1*-*ter1* ratio was always in favour of *oriC1*, even reaching a point of 6 *oriC1* to 1 *ter1* (panel 225’). In the absence of *matP*, duplicated *ter1* foci had the tendency to remain apart from each other after segregation and the number of *ter1* foci closely followed the number of *oriC1*, from 2 to 4 to 8 distinct individual foci (Fig 5B and S26 Movie).

Together these results suggest that MatP was able to keep together sister *ter1* regions independently of cell shape and proliferation.

### The polar determinant HubP does not originate cell branching

**Fig 6:**
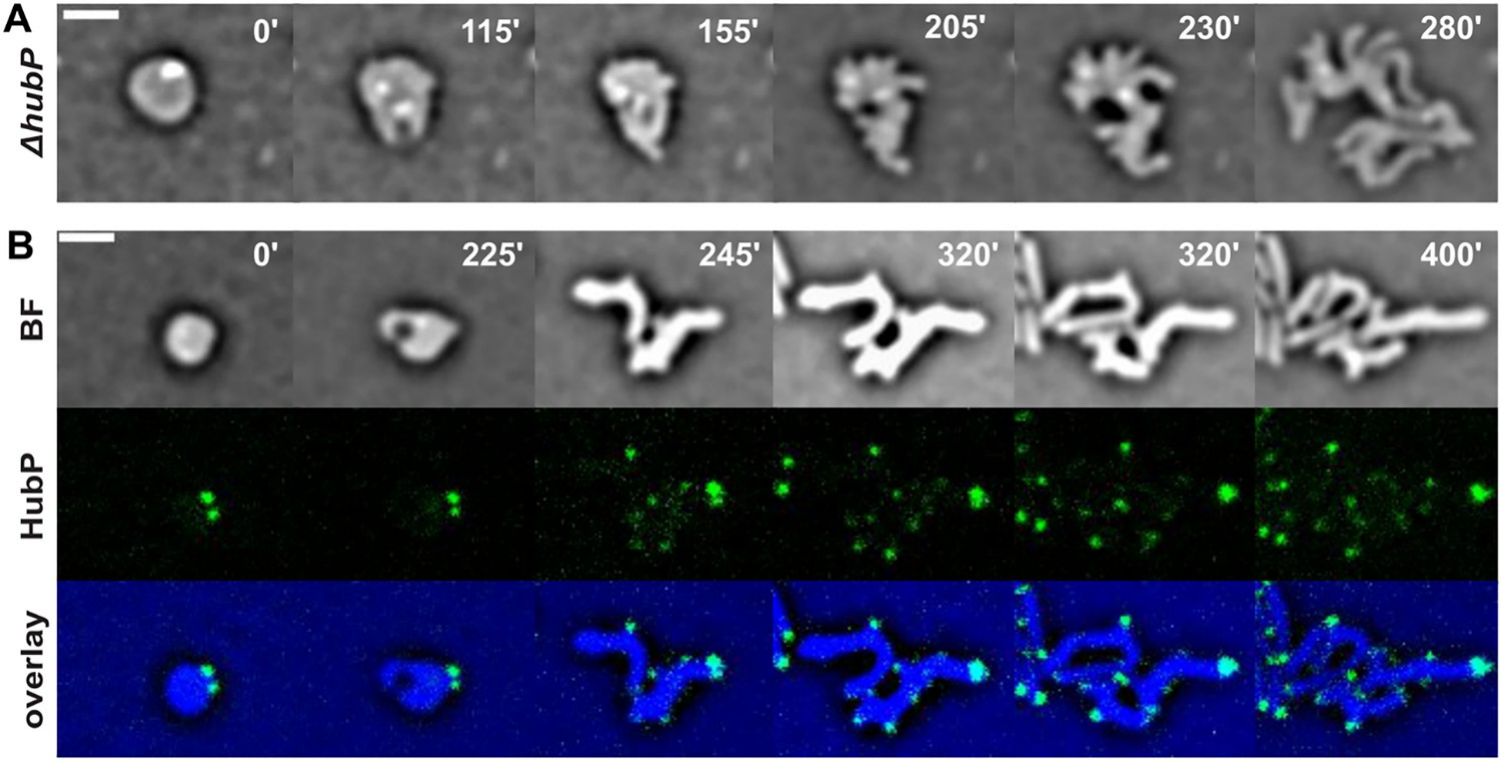
Branching formation during cell shape recovery. Reconstructed time-lapse BF and fluorescent images. N16961 derivative cells were grown on M9-MM agarose pads at 30°C. One frame was taken every 5 minutes. On the top-right corner of each frame is indicated the time in minutes. Scale bars = 2 μm. **A.** Branching formation in Δ*hubP* recovering cells. **B.** Localization of HubP-sfGFP.

Formation of outward bulges branching off elongating spherical cells and subsequently growing into rods is a common characteristic of morphogenetic processes [37, 38, 49]. It is still unknown how these branches originate and what locally characterizes their nucleation site on the cell surface. We tested if the polar determinant HubP [16, 30] was involved in marking the site from which bulges emerged by following the recovery process of Δ*hubP* cells at the single-cell level on agarose pads. All steps of sphere elongation, periplasmic engulfment and branching were clearly visible rendering the sphere to rod transition indistinguishable from wild-type cells (Fig 6A and S27 Movie). To determine if HubP targeted the nascent poles, we followed the localization pattern of a fully functional HubP-sfGFP fluorescent fusion protein (Fig 6B and S28 Movie). In spherical cells, HubP always localized as 2 foci at the periphery of the cell (Fig 6A, panel 0’), characteristically never positioned at the site of the periplasmic excess. During the entire recovery process, the 2 original HubP foci appeared static and never moved from their original position. In what it seemed a passive process, they were first located at the polar region of the elongating sphere and in the end at the pole of one of the rod-shaped progenies. On the contrary, HubP dynamically accumulated at all newly formed poles, originated either by branching or by a cell division event.

## Discussion

Bacteria can withstand or even overcome a variety of stresses, such as nutrient deprivation or antibiotic treatment. In particular, *V*. *cholerae* is able to halt proliferation and reduce its cellular metabolism until growth conditions are restored, at which point it reverts to vegetative growth. Non-proliferating *V*. *cholerae* cells adopt a spherical shape, which is characterized by a depletion of the cell-wall material and the loss of normal polarized cell organization [27, 28]. Resumption of proliferation requires the synthesis and insertion of new cell-wall material and the recovery of a polarized organization of all cellular machineries.

### Rod shape recovery

The observation by fluorescent video-microscopy of hundreds of L-Ara-induced *V*. *cholerae* spheroplasts returning to growth on agarose pads revealed that each spheroplast produced multiple rod-shaped cells. The standard recovery pattern began with a periplasmic engulfment step in which an excess of periplasm on one side of the spheroplasts was engulfed and then extruded in the form of vesicles. This was followed by cell elongation, the formation of outgrowing cell branches with a diameter similar to that of rod-shaped *V*. *cholerae* and multiple cell division events (Fig 1 and S1–S3 Movies). We found that the bifunctional PG synthases aPBPs played an essential role in the periplasmic engulfment stage whereas the Rod-complex machinery was required for cell elongation and branching (Fig 2A and S12 Movie), as suggested for spherical cell-wall deficient cells formed after treatment with cell-wall targeting antibiotics [49]. The Rod-complex was not essential for the elimination of the periplasmic excess. However, the engulfment process took a longer time to be accomplished in conditions that inhibited the activity of MreB or PBP2, suggesting that it helped the process (Fig 2CD and S16–S17 Movies). The divisome was only involved in cytokinesis (Fig 2E and S18 Movie).

The roles of the different cell-wall synthetic machineries during the *de novo* morphogenesis were confirmed by monitoring the localization of selected fluorescently labelled PBPs and cell division factors. PBP1a, whose inhibition impeded periplasmic engulfment, localized at the inner edge of the periplasmic excess and encircled it during the engulfment process (Fig 2B and S13 Movie) whereas the Z-ring components, FtsZ and FtsI, whose inactivation did not impede formation of filamentous rods (Fig 2E and S18 Movie), only localized at sites of cell constriction and septum formation (Fig 3AB and S19–S20 Movies). In vegetatively growing cells, all cell division factors colocalize with FtsZ [16]. In contrast, in rod-shape recovering cells, FtsK localized to the leading edge of the periplasmic engulfment and to sites of periplasmic vesicle extrusion independently of FtsZ (Fig 3C and S21 Movie). This observation suggests that FtsK may be involved in the membrane fusion events that lead to the elimination of outer membrane vesicles. Consistently with this hypothesis, FtsK has previously been proposed to facilitate membrane fusion during cytokinesis in *E*. *coli* [53] and a *B*. *subtilis* homologue of FtsK, SpoIIIE, was proposed to be involved in membrane fusion following spore engulfment [54, 55].

The standard recovery pattern of L-Ara-induced *V*. *cholerae* spheroplasts differs markedly from that of *E*. *coli* spheroplasts induced by treatment with lysozyme or cefsulodin. *E*. *coli* spheroplasts do not show a periplasmic excess. Therefore, there is no periplasmic engulfment step. The recovery process of cefsulodin-induced *E*. *coli* spheroplasts was similar to that of the 6% of L-Ara-induced *V*. *cholerae* spheroplasts that failed to undergo periplasmic engulfment, in which new rod-shaped cells are generated by the formation and elongation of a tubular protrusion by the Rod-complex, and subsequent division events (Fig 1B, [37, 38]). The recovery process of lysozyme-induced *E*. *coli* spheroplasts was similar to that of the 6.5% of L-Ara-induced spheroplasts that did not undergo periplasmic engulfment, in which rod-shaped cells are generated by elongation of the spheroplast and subsequent division events (Fig 1C, [37, 38]). Differences in the recovery patterns of cefsulodin- and lysozyme-induced *E*. *coli* spheroplasts have been proposed to result from differences in PG content, with cefsulodin-induced spheroplasts retaining PG remnants and glycan strands whereas lysozyme-induced spheroplasts have no pre-existing PG [56]. This suggested the possibility that the 3 recovery patterns observed for the L-Ara-induced *V*. *cholerae* spheroplasts were due to differences in their PG content. However, we were able to follow the recovery of some L-Ara-induced *V*. *cholerae* spheroplasts in microfluidic chambers (S29 Movie and S2 Fig). All of the observed spheroplasts contained an elongating bulge juxtaposed the periplasmic excess site and followed a recovery pattern resembling that of L-Ara-induced *V*. *cholerae* spheroplasts that failed to complete periplasmic engulfment on agarose pads (S4–S5 Movies). This result suggests that the ability to complete periplasmic engulfment was related to differences in growth conditions rather than to the PG content of the cells, in agreement with the rod shape recovery patterns of *V*. *cholerae* spheroplasts induced by treatment with cell-wall targeting antibiotics [49].

### Chromosome organization recovery

L-Ara-treated spheroplasts contained fully replicated Chr1 and Chr2 copies, with the vast majority of cells carrying two complete sets of chromosomes (S1 Fig). This is reminiscent of *V*. *cholerae* cells under nutrient starvation stress, where the stringent response blocks the initiation of new rounds of DNA replication while ongoing rounds are terminated [57]. The simultaneous appearance of two SeqA foci early in the recovery process suggested synchronous replication initiation of the two Chr1 copies (Fig 4A). Consistent with *crtS*-dependent replication initiation of Chr2 copies [52], SeqA foci increased from 2 to 4 soon after replication restarted and doubling of the origin of replication of Chr1 and Chr2 were temporally separated (Fig 4AB and S22 and S23 Movies). The replication rounds proceeded in synchronous waves, with SeqA foci disappearing and reappearing in an orderly fashion in doubled numbers (Fig 4A and S22 Movie). It is also worth noting that SeqA foci, which initially appeared very bright, soon faded slightly in intensity and split into two adjacent foci, as if a replication complex controlled by SeqA was present on the left and right replichore of each chromosome during DNA replication (S22 Movie). This suggests that there are no replisome factories on either Chr1 or Chr2 in *V*. *cholerae*, as in *E*. *coli* [58].

The polar marker HubP localized at the pole of the branches that appeared during recovery, independently of division (Fig 6B and S28 Movie). HubP is required for the polar placement of *oriC1* [30]. In its absence, *oriC1* polar anchoring is lost and chromosomes display a ParAB1-dependent transversal organization in rods [31]. Yet, we did not observe any proliferation arrest, filamentation or lysis in the progeny formed by branching and subsequent division events in Δ*hubP* recovering cells (Fig 6A and S27 Movie). This suggests that chromosomes were correctly segregated into the elongating branches in the absence of HubP.

The *oriC1* and *oriC2* choreographies further showed that chromosomes tended to occupy all the available cell volume even before the onset of replication and morphogenetic reversion to rods. After duplication, in cells that were still spherical, the replication origins separated from each other and appeared very mobile. As the recovering cells began to elongate or branch, they aligned themselves with the longitudinal axes of the developing rods (Fig 4B and S23 Movie). This was particularly evident in the choreography of the Chr1 segregation factor ParB1, which localized to the newly formed cellular protrusions that eventually branched out and elongated into rods (Fig 4C and S24 Movie).

In contrast to *oriC1* loci, duplicated *ter1* loci remained together in spherical cells. In addition, newly replicated sister *ter1* loci remained together for an extended period of time during recovery, with only transient separations (Fig 5A and S25 Movie). The cohesiveness of *ter1* foci depended on MatP (Fig 5B, S1 Fig and S26 Movie). In *E*. *coli*, MatP binds to specific motifs, the *matS* sites, which are located in the chromosome replication termination regions (Ter). It interacts with the cell division machinery via the ZapB-ZapA-FtsZ protein chain, thereby maintaining sister Ter regions at midcell by linking them to the Z-ring [59, 60]. However, our results suggest that *V*. *cholerae* MatP keeps sister Ter regions together independently of the Z-ring since it is absent in the L-Ara-induced *V*. *cholerae* spheroplasts and during the early stages of recovery.

## Material and methods

### Plasmids and strains

Bacterial strains, plasmids, protein fusion linkers and primers used in this study are listed in S1 Table. All strains are derivatives of the El Tor N16961 strain rendered competent by the insertion of *hapR* by specific transposition and constructed by natural transformation or conjugation with integration/excision plasmids. Plasmids and strains construction is described in detail in S1 File. Engineered strains were confirmed by PCR.

The *ftsK-YGFP*, *hubP-sfGFP*, *seqA-YGFP* and *PBP1a-sfGFP* fusions were inserted in place of the endogenous *V*. *cholerae ftsK*, *hubP*, *seqA* and *PBP1a* allele, respectively. All other fusion genes were introduced at the *lacZ* or *hapR* locus in addition to the wild-type allele. *lacI-RFPT*, *YGFP-parB*^*pMT1*^, *YGFP-parB1*, *YGFP-ftsI* and *ftsZ-RFPT* fluorescent protein fusions were produced from the *E*. *coli lacZ* promoter, leakiness of the promoter was sufficient for imaging. *ftsZ-RFPT* starting codon was changed from ATG to TTG to reduce expression and avoid cell filamentation originated by FtsZ excess.

*oriC1*, *oriC2* and *ter1* chromosomal loci were visualized by inserting a pMT1 *parS* motif next to *oriC1* and a *lacO* array next to *oriC2* or *ter1* that were specifically detected by LacI-RFPT and YGFP-ParB^pMT1^, respectively.

The cytoplasm was visualized by expressing YGFP from an *E*. *coli lacZ* promoter in the cytoplasm and the periplasm by expression from a Zn^2+^-inducible promoter [61] of mCherry fused to the DsbA signal sequence to efficiently export it to the periplasm [62]. In both cases, leakiness of the promoter was sufficient for visualization.

### Growth conditions

If not otherwise indicated, cells were grown at 30°C in M9 minimal medium supplemented with 0.2% (wt/vol) fructose and 1 mg/ml (wt/vol) thiamine (M9-MM). Overnight cultures were diluted 100 times in M9-MM (to an OD600 0.02), followed by 2 hours of growth at 30°C before 0.2% (wt/vol) L-Ara was added. Cells were incubated shacking at 30°C for further 7h to 10h before cells were washed in L-Ara-free medium and spread on agarose pads or collected for further analyses. The antibiotics used and their final concentration were the following: 1 mg/ml (wt/vol) cefsulodin, 10 μg/ml (wt/vol) A22, 10 μg/ml (wt/vol) mecillinam.

### Microscopy

For standard microscopy experiments, cells were spread on a 1% (wt/vol) agarose pad made using M9-MM. For time-lapse analyses in liquid medium, we injected L-Ara treated cells in a PDMS microfluidic device with 1 μm deep micro-chambers. A syringe pump is used to inject fresh M9-MM that by diffusion enters the micro-chambers for a constant supply of fresh medium. For snapshots, images were acquired using a DM6000-B (Leica) microscope. For time-lapse analyses, images were acquired using an Evolve 512 EMCCD camera (Roper Scientific) attached to an Axio Observe spinning disk (Zeiss). If needed, antibiotics were added to the M9-MM agarose pads. Pictures were taken every 5 minutes. At each time point, we took a stack of 32 bright-field images covering positions 1.6 μm below and above the focal plane. The final single BF image was reconstructed using a MatLab-based script developed in the lab [24]. The fluorescent image, if needed, was taken only once, at the focal plane, to avoid photobleaching.

### Marker frequency analysis by whole-genome sequencing

Samples were collected before L-Ara addition (T0) and after a 10h (T10) incubation with 0.2% (wt/vol) L-Ara. Chromosomal DNA was extracted using the Sigma GenElute® bacterial genomic DNA kit to generate a genomic library according to Illumina’s protocol. The libraries and the sequencing were performed by the High-throughput Sequencing facility of the I2BC (CNRS, Gif-sur-Yvette, France). Genomic DNA libraries were made with the ‘Nextera DNA library preparation kit’ (Illumina) following the manufacturer’s recommendations. Library quality was assessed on an Agilent Bioanalyzer 2100, using an Agilent High Sensitivity DNA Kit (Agilent technologies). Libraries were pooled in equimolar proportions. 75 bp single reads were generated on an Illumina MiSeq instrument, using a MiSeq Reagent kit V2 (500 cycles) (Illumina), with an expected depth of 217X. Marker frequency analysis was performed using a MatLab-based script developed in the lab [36]. The FASTQ files of the reads have been deposited in the NCBI SRA database (Accession number PRJNA1027307).

### Flow cytometry

Samples were collected before L-Ara addition (T0) and after a 5h (T5) and 7h (T7) incubation with 0.2% (wt/vol) L-Ara and fixed in 70% ethanol overnight at 4°C. Samples were washed twice in TE buffer pH 7.5 (10 mM Tris, 1 mM EDTA) and resuspended in 100 μl TE buffer + 10 μg/ml (wt/vol) RNase A + 10 μg/ml (wt/vol) propidium iodide and incubated 1h at 37°C. Stained cells were analyzed on a Partec PAS III, typically 100 000 cells were analyzed in each run and data were analyzed with Flow max v. 2.52.

## Supporting information

S1 Fig **A.** DNA histograms of chromosome sets per cell obtained by flow cytometry. N16961 derivative cells (strain EPV50) were grown in M9-MM at 30°C and samples collected before L-Ara addition (T0) and after a 5h (T5) and 7h (T7) incubation with L-Ara. **B.** Marker frequency analysis profile of N16961 derivative cells (strain EPV50) grown in M9-MM at 30°C before L-Ara addition (T0) and after a 10h incubation with L-Ara (T10). **C.** Number of chromosome sets per cell obtained by counting the number of *oriC1* and *oriC2* foci in N16961 derivative cells (strain EGV346) incubated for 7h with L-Ara in M9-MM at 30°C. Mean of two independent replicates (~1000 cells each) and the standard deviation are represented. **D.** Percentage of cells with 2 *oriC1* foci vs 1 *ter1* focus and 2 *oriC1* foci vs 2 *ter1* foci in wild-type (strain EGV324) and Δ*matP* (strain EGV326) cells incubated for 7h with L-Ara in M9-MM at 30°C. Mean of two independent replicates (~1000 cells each) and the standard deviation are represented.
(TIF)

S2 Fig Reconstructed time-lapse bright-field (BF) images of *V*. *cholerae* N16961 derivatives cells grown at 30°C in a microfluidic device supplemented with M9-MM after L-Ara removal. One frame was taken every 5 minutes. On the top-right corner of each frame is indicated the time in minutes. Scale bar = 5 μm. The stars point to the periplasmic excess and the arrows to the elongating rod-shaped bulge juxtaposed to the periplasmic excess.
(TIF)

S1 Movie Recovery of rod shape with a periplasmic engulfment process. One frame was taken every 5 minutes. N16961 derivative cells were grown in M9-MM at 30°C. The periplasm is visible by periplasmic mCherry diffusion (red) and the cytoplasm by cytoplasmic YGFP diffusion (green).
(AVI)

S2 Movie Recovery of rod shape with a periplasmic engulfment process. One frame was taken every 5 minutes. N16961 derivative cells were grown in M9-MM at 30°C. The periplasm is visible by periplasmic mCherry diffusion (red) and the cytoplasm by cytoplasmic YGFP diffusion (green).
(AVI)

S3 Movie Recovery of rod shape with a periplasmic engulfment process. One frame was taken every 5 minutes. N16961 derivative cells were grown in M9-MM at 30°C. The periplasm is visible by periplasmic mCherry diffusion (red).
(AVI)

S4 Movie Recovery of rod shape with a failed periplasmic engulfment process. One frame was taken every 5 minutes. N16961 derivative cells were grown in M9-MM at 30°C.
(AVI)

S5 Movie Recovery of rod shape with a failed periplasmic engulfment process. One frame was taken every 5 minutes. N16961 derivative cells were grown in M9-MM at 30°C. The periplasm is visible by periplasmic mCherry diffusion (red).
(AVI)

S6 Movie Recovery of rod shape with a failed periplasmic engulfment process. One frame was taken every 5 minutes. N16961 derivative cells were grown in M9-MM at 30°C.
(AVI)

S7 Movie Transition to spherical cells in presence of 0.2% L-Ara. One frame was taken every 5 minutes. N16961 derivative cells were grown in M9-MM supplemented with 0.2% L-Ara at 30°C. The periplasm is visible by periplasmic mCherry diffusion (red).
(AVI)

S8 Movie Recovery of rod shape without a periplasmic engulfment process. One frame was taken every 5 minutes. N16961 derivative cells were grown in M9-MM at 30°C.
(AVI)

S9 Movie Recovery of rod shape without a periplasmic engulfment process. One frame was taken every 5 minutes. N16961 derivative cells were grown in M9-MM at 30°C.
(AVI)

S10 Movie Recovery of rod shape without a periplasmic engulfment process. One frame was taken every 5 minutes. N16961 derivative cells were grown in M9-MM at 30°C.
(AVI)

S11 Movie Time-lapse of exponentially growing N16961 derivative cells (strain EPV50) grown in M9-MM + 1 mg/ml cefsulodin at 30°C. One frame was taken every 5 minutes.
(AVI)

S12 Movie Failed return to rod shape of spherical cells treated with cefsulodin. One frame was taken every 5 minutes. N16961 derivative cells (strain EPV50) were grown in M9-MM + 1 mg/ml cefsulodin at 30°C.
(AVI)

S13 Movie Localization of PBP1a-sfGFP during recovery of rod shape. One frame was taken every 5 minutes. N16961 derivative cells (strain EGV623) were grown in M9-MM at 30°C.
(AVI)

S14 Movie Time-lapse of exponentially growing N16961 derivative cells (strain EPV50) grown in M9-MM + 10 μg/ml A22 at 30°C. One frame was taken every 5 minutes.
(AVI)

S15 Movie Time-lapse of exponentially growing N16961 derivative cells (strain EPV50) grown in M9-MM + 10 μg/ml mecillinam at 30°C. One frame was taken every 5 minutes.
(AVI)

S16 Movie Failed return to rod shape of spherical cells treated with A22. One frame was taken every 5 minutes. N16961 derivative cells (strain EPV50) were grown in M9-MM + 10 μg/ml A22 at 30°C.
(AVI)

S17 Movie Failed return to rod shape of spherical cells treated with mecillinam. One frame was taken every 5 minutes. N16961 derivative cells (strain EPV50) were grown in M9-MM + 10 μg/ml mecillinam at 30°C.
(AVI)

S18 Movie Recovery of rod shape of N16961 derivative cells carrying the *ftsZ84* temperature sensitive mutation (strain EPV390). One frame was taken every 5 minutes. Cells were grown in M9-MM at 42°C.
(AVI)

S19 Movie Localization of FtsZ-RFPT during recovery of rod shape. One frame was taken every 5 minutes. N16961 derivative cells (strain AGV4) were grown in M9-MM at 30°C.
(AVI)

S20 Movie Localization of YGFP-FtsI during recovery of rod shape. One frame was taken every 5 minutes. N16961 derivative cells (strain EGV9) were grown in M9-MM at 30°C.
(AVI)

S21 Movie Localization of FtsK-YGFP during recovery of rod shape. One frame was taken every 5 minutes. N16961 derivative cells (strain EGV34) were grown in M9-MM at 30°C.
(AVI)

S22 Movie Localization of SeqA-YGFP during recovery of rod shape. One frame was taken every 5 minutes. N16961 derivative cells (strain AHV42) were grown in M9-MM at 30°C.
(AVI)

S23 Movie Choreographies of *oriC1* and *oriC2* loci during recovery of rod shape. *oriC1* foci are in green and *oriC2* foci in red. One frame was taken every 5 minutes. N16961 derivative cells (strain EGV346) were grown in M9-MM at 30°C.
(AVI)

S24 Movie Localization of YGFP-ParB1 during recovery of rod shape. One frame was taken every 5 minutes. N16961 derivative cells (strain EGV72) were grown in M9-MM at 30°C.
(AVI)

S25 Movie Choreographies of *oriC1* and *ter1* loci during recovery of rod shape. *oriC1* foci are in green and *ter1* foci in red. One frame was taken every 5 minutes. N16961 derivative cells (strain EGV324) were grown in M9-MM at 30°C.
(AVI)

S26 Movie Choreographies of *oriC1* and *ter1* loci during recovery of rod shape in a Δ*matP* background. *oriC1* foci are in green and *ter1* foci in red. One frame was taken every 5 minutes. N16961 derivative cells (strain EGV326) were grown in M9-MM at 30°C.
(AVI)

S27 Movie Recovery of rod shape with formation of branches in a Δ*hubP* background. One frame was taken every 5 minutes. N16961 derivative cells (strain EGV75) were grown in M9-MM at 30°C.
(AVI)

S28 Movie Localization of HubP-sfGFP during recovery of rod shape. One frame was taken every 5 minutes. N16961 derivative cells (strain EPV453) were grown in M9-MM at 30°C.
(AVI)

S29 Movie Recovery of rod shape in a microfluidic PDMS device in M9-MM at 30°C. One frame was taken every 5 minutes.
(AVI)

S1 File Detailed description of plasmids and bacterial strains construction. (DOCX)

S1 Table List of bacterial strains, plasmids, protein linkers and primers used in this study. (DOCX)
