# Supplementary figures and images for: Recovery of Vibrio cholerae polarized cellular organization after exit from a non-proliferating spheroplast state
Source: PLoS One. 2023 Oct 26;18(10):e0293276. doi: 10.1371/journal.pone.0293276 (PMC10602287; doi:10.1371/journal.pone.0293276)

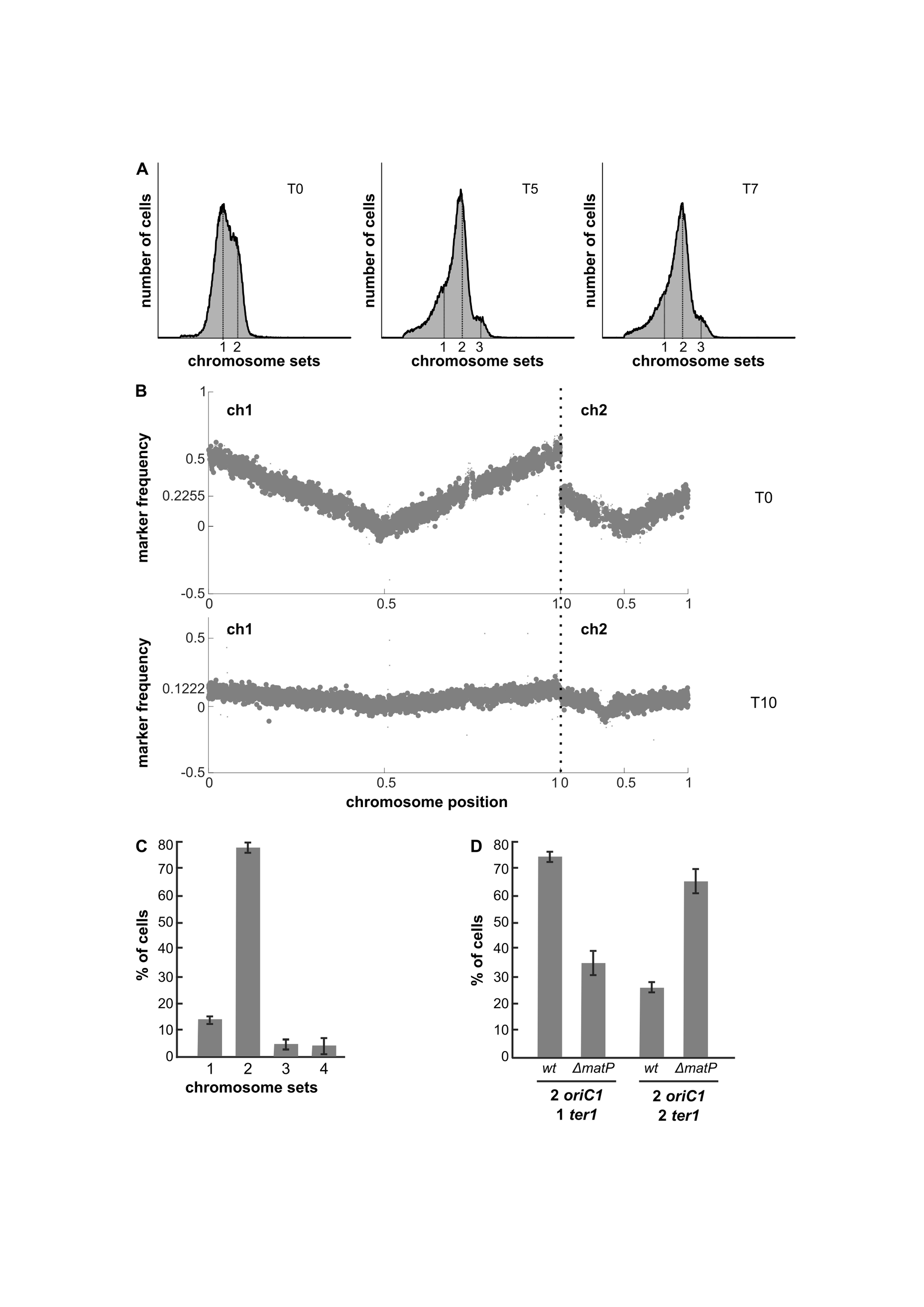

Supplement: S1 Fig — A. DNA histograms of chromosome sets per cell obtained by flow cytometry. N16961 derivative cells (strain EPV50) were grown in M9-MM at 30°C and samples collected before L-Ara addition (T0) and after a 5h (T5) and 7h (T7) incubation with L-Ara. B. Marker frequency analysis profile of N16961 derivative cells (strain EPV50) grown in M9-MM at 30°C before L-Ara addition (T0) and after a 10h incubation with L-Ara (T10). C. Number of chromosome sets per cell obtained by counting the number of oriC1 and oriC2 foci in N16961 derivative cells (strain EGV346) incubated for 7h with L-Ara in M9-MM at 30°C. Mean of two independent replicates (~1000 cells each) and the standard deviation are represented. D. Percentage of cells with 2 oriC1 foci vs 1 ter1 focus and 2 oriC1 foci vs 2 ter1 foci in wild-type (strain EGV324) and ΔmatP (strain EGV326) cells incubated for 7h with L-Ara in M9-MM at 30°C. Mean of two independent replicates (~1000 cells each) and the standard deviation are represented. (TIF) [file pone.0293276.s001.tif]

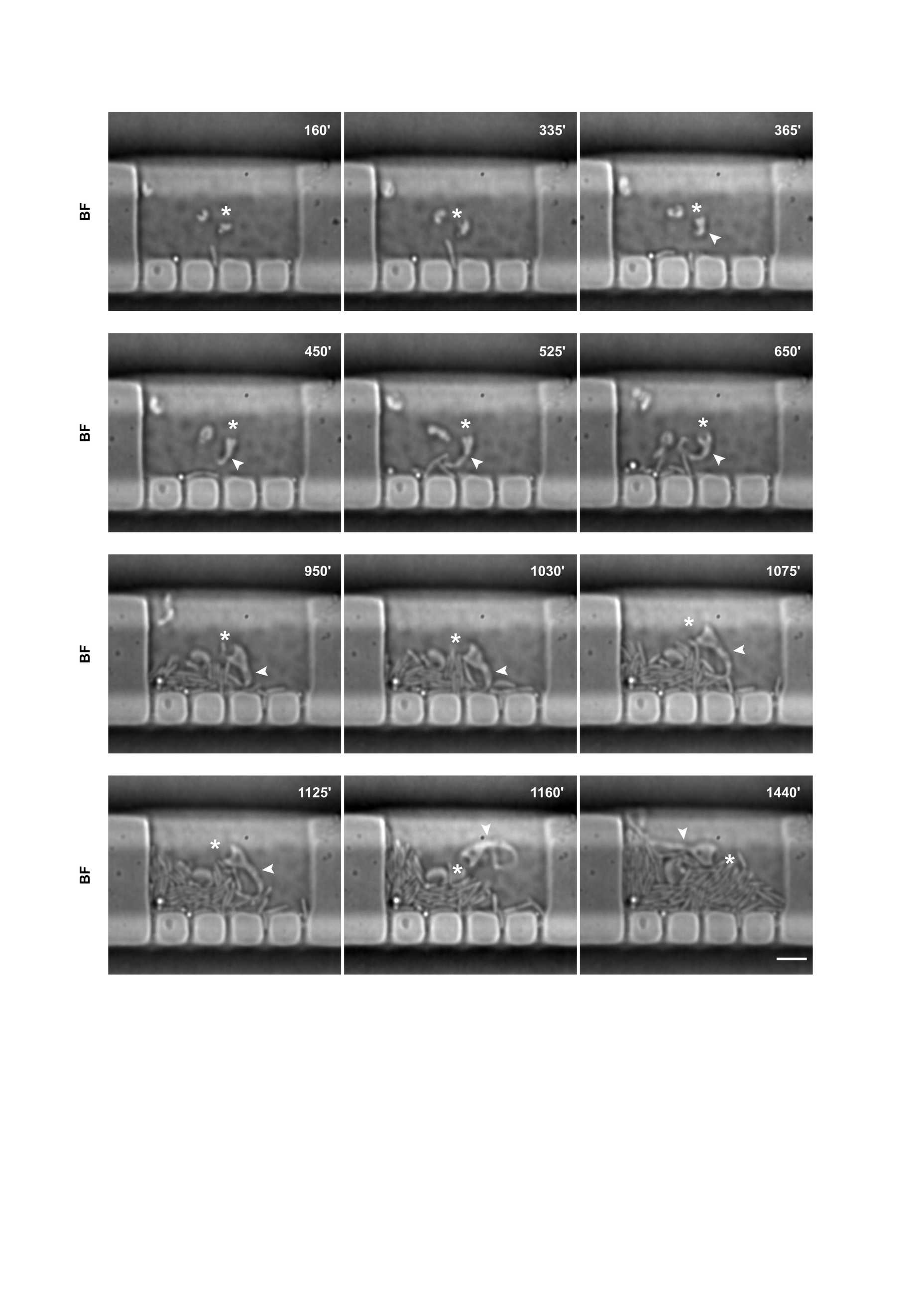

Supplement: S2 Fig — One frame was taken every 5 minutes. On the top-right corner of each frame is indicated the time in minutes. Scale bar = 5 μm. The stars point to the periplasmic excess and the arrows to the elongating rod-shaped bulge juxtaposed to the periplasmic excess. (TIF) [file pone.0293276.s002.tif]
