# Supplementary material for: Recovery of Vibrio cholerae polarized cellular organization after exit from a non-proliferating spheroplast state
Source: PLoS One. 2023 Oct 26;18(10):e0293276. doi: 10.1371/journal.pone.0293276 (PMC10602287; doi:10.1371/journal.pone.0293276)
Supplement: S1 File — (DOCX) [file pone.0293276.s032.docx]

**Construction of plasmids**


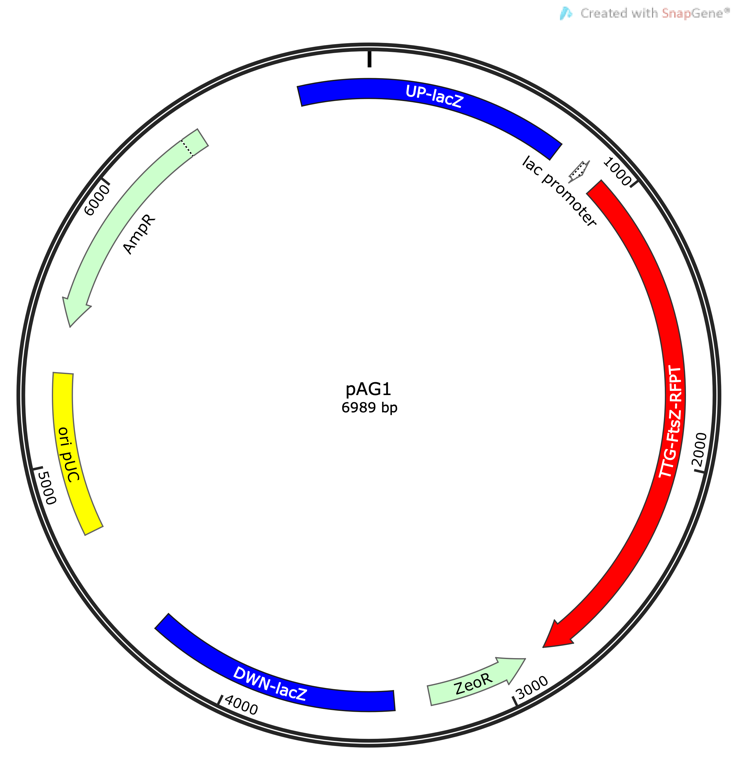
**pAG1** carries *TTG-ftsZ-RFPT* downstream of the P*_lac_* promoter to be inserted in *lacZ*. The *TTG-ftsZ* gene was amplified with primers 4293+2502 from N16961 chromosomal DNA, digested with XhoI-HindIII and cloned into the corresponding sites of pEP76 (laboratory collection), replacing *lacI* in the P*_lac_*::*lacI-RFPT* fusion. UP=upstream; DWN=downstream. Used for natural transformation.

**pAG1 map:**

Integration site: *lacZ* (in blue)

Integrated sequence: P*_lac_::ftsZ-RFPT*, ZeoR

Clone selection after natural transformation: ZeoR

**pAH3** carries *seqA-YGFP* downstream of the *seqA* native promoter to be inserted at *seqA* native locus. The plasmid was constructed by Gibson Assembly mixing together 5 PCR products. PCR fragment 1 was amplified with primers 542+2896 from pFX497 (laboratory collection) and carries the plasmid pSC101 origin of replication and the *bla* gene for AmpR. PCR fragment 2 was amplified with primers 2897+2898 from N16961 chromosomal DNA and carries the upstream sequence of *seqA* and the *seqA* gene with its own promoter. PCR fragment 3 was amplified with primers 2899+2900 from pEG248 [1] and carries the *YGFP* gene. PCR fragment 4 was amplified with primers 2901+2895 from pEP70 [2] and carries the *Sh ble* gene for ZeoR. PCR fragment 5 was amplified with primers 2902+2903 from N16961 chromosomal DNA and carries the downstream region of *seqA*. Used for natural transformation.


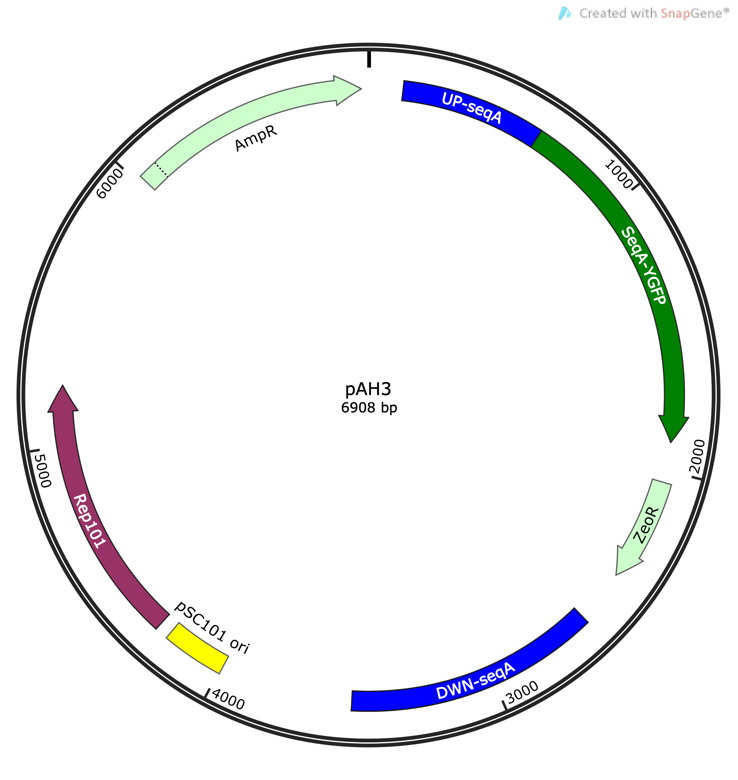


**pAH3 map:**

Integration site: *seqA* (in blue)

Integrated sequence: P*_seqA_::seqA-YGFP*, ZeoR

Clone selection after natural transformation: ZeoR


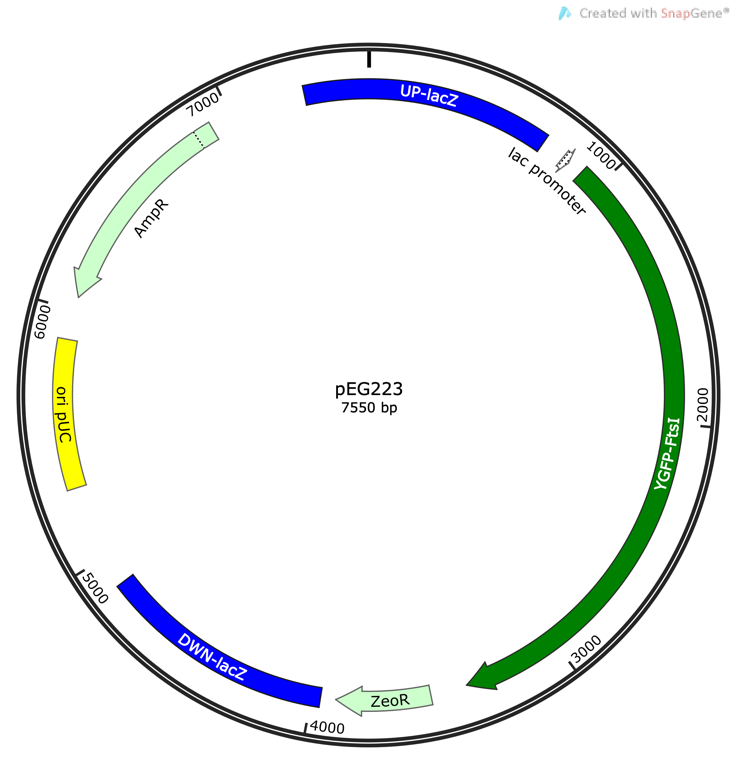
**pEG223** carries *YGFP-ftsI* downstream of the P*_lac_* promoter to be inserted in *lacZ*. The *ftsI* gene was amplified by PCR with primers 2509+2510 from N16961 chromosomal DNA, digested with SphI-SpeI and cloned into the corresponding sites of pEP57 (laboratory collection), replacing *parB^pMT1^* in the P*_lac_*::*YGFP-parB^pMT1^* fusion. Used for natural transformation.

**pEG223 map:**

Integration site: *lacZ* (in blue)

Integrated sequence: P*_lac_::YGFP-ftsI*, ZeoR

Clone selection after natural transformation: ZeoR


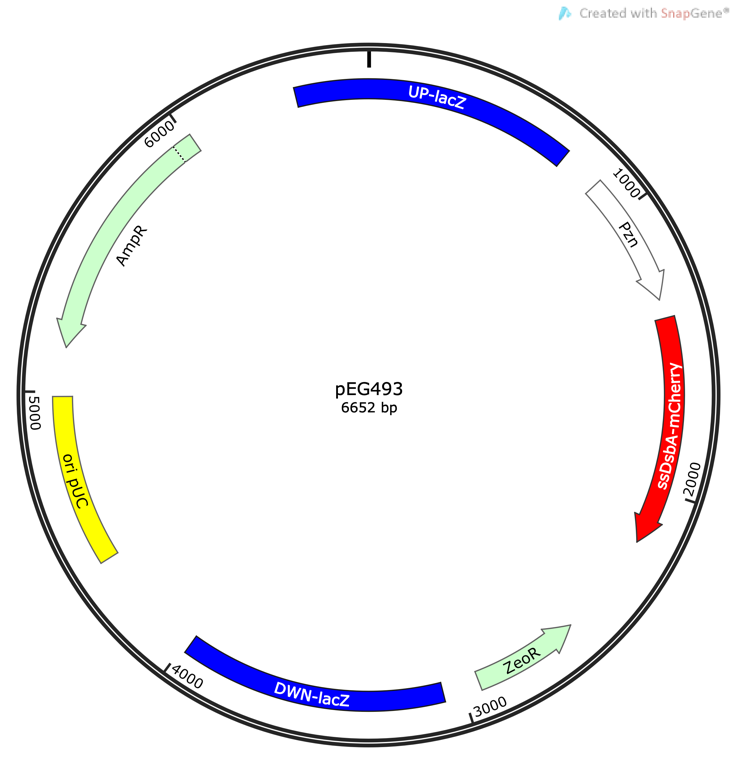
**pEG493** carries *dsbA*_ss_*-mCherry* downstream of the P*_zn_* promoter to be inserted in *lacZ*. At first we constructed plasmid pEG444 by inserting the P*_zn_* promoter of pMK17-01 [3] in place of P*_lac_::lacI-RFPT-parB^pMT1^-YGFP* of pEP70 [2] by Gibson assembly. PCR fragment 1 was amplified with primers 526+4002 from pEP70 and carries the plasmid pUC origin of replication and the *bla* gene for AmpR. PCR fragment 2 was amplified with primers 4005+4006 from pMK17-01 and carries the P*_zn_* promoter. Then, we constructed the *dsbA*_ss_*-mCherry* fusion in plasmid pEG486 by replacing *PAmCherry* of pEYY225 [4] *dsbA*_ss_*-PamCherry* with *mCherry*. The *mCherry* gene was amplified by PCR with primers 2147+4229 from pEG392 [5], digested with XhoI-HindIII and cloned into the corresponding sites of pEYY225. The NdeI-HindIII fragment of pEG486 carrying *dsbA*_ss_*-mCherry* was subcloned into the corresponding sites of pEG444 downstream of the P*_zn_* promoter, completing pEG493 construction. Used for natural transformation.

**pEG493 map:**

Integration site: *lacZ* (in blue)

Integrated sequence: P*_zn_::dsbA_ss_-mCherry*, ZeoR

Clone selection after natural transformation: ZeoR


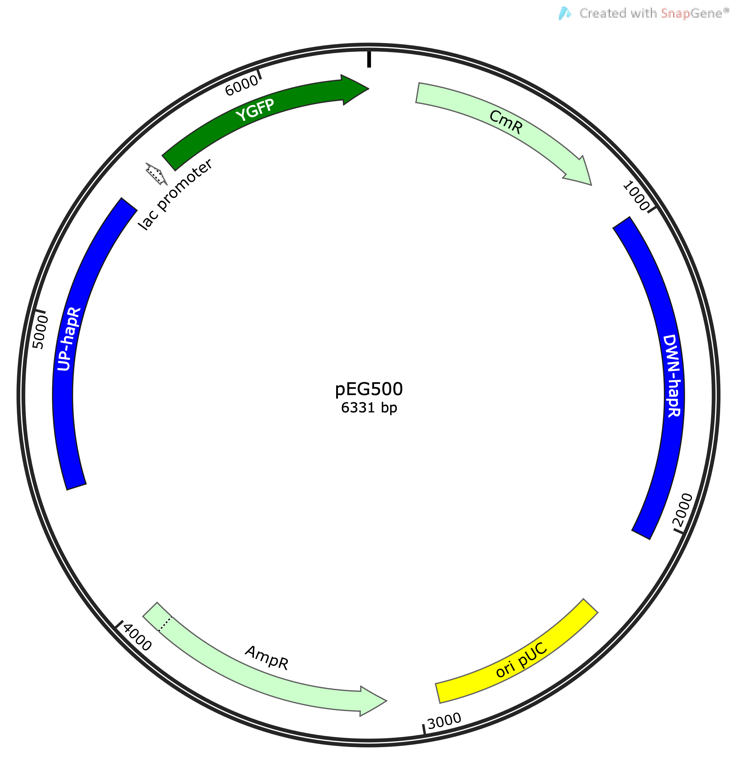
**pEG500** carries *YGFP* downstream of the P*_lac_* promoter to be inserted in *hapR*. The *YGFP* gene was amplified with primers 2296+3707 from pEG245 [5], digested with NdeI(blunted)-SpeI and cloned into the AleI-SpeI sites of pEG246 [1]. Used for natural transformation.

**pEG500 map:**

Integration site: *hapR* (in blue)

Integrated sequence: P*_lac_::YGFP*, CmR

Clone selection after natural transformation: CmR

**pEG504** carries *PBP1a-sfGFP* downstream of the *PBP1a* native promoter to be inserted at *PBP1a* native locus. We first constructed by Gibson assembly plasmid pEG499 carrying *PBP1a-mCherry*. PCR fragment 1 was amplified with primers 3331+3340 from pEYY24 (gift from Y. Yamaichi laboratory) and carries the plasmid R6K origin of replication, the *bla* gene for AmpR and the *sacB* gene. PCR fragment 2 was amplified with primers 4296+4297 from N16961 chromosomal DNA and carries the *PBP1a* gene. PCR fragment 3 was amplified with primers 4273+4290 from pEG392 [5] and carries the *mCherry* gene. PCR fragment 4 was amplified with primers 4298+4299 from N16961 chromosomal DNA and carries the downstream region of *PBP1a*. Subsequently, *mCherry* was replaced by *sfGFP* creating plasmid pEG504. The *sfGFP* gene was amplified with primers 2297+4271 from pEYY24, digested with XhoI-SpeI and cloned into the corresponding sites of pEG499. Used for integration/excision.


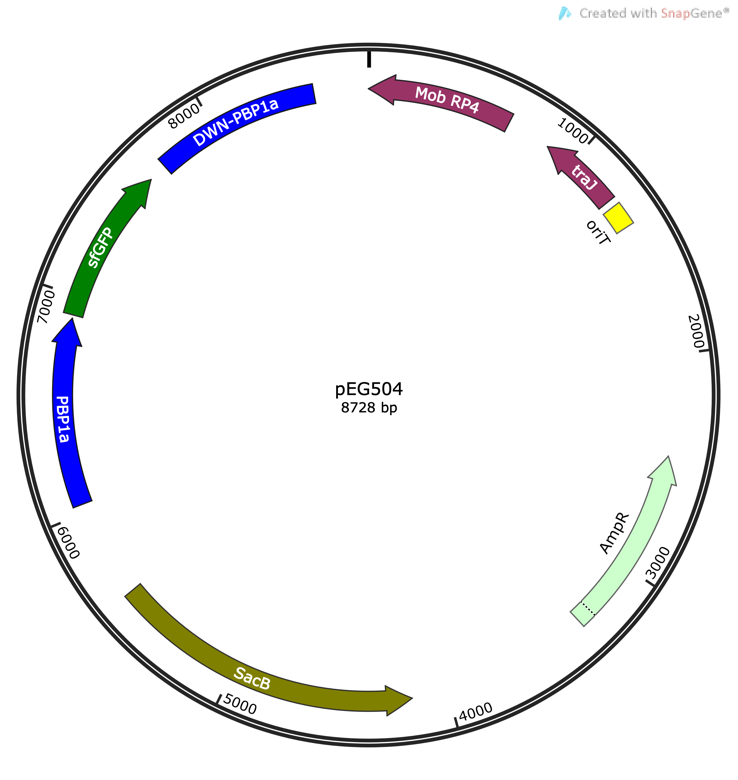


**pEG504 map:**

Integration site: *PBP1a* (in blue)

Integrated sequence: P*_PBP1a_::PBP1a-sfGFP*

Plasmid integration selection: AmpR

Plasmid excision selection: AmpS

**Construction and verification of strains**

All strains are derivatives of EPV50 (El Tor N16961 rendered competent by insertion of *hapR*) [6]. All strains were constructed by integration-excision or natural transformation, a 700bp-1Kbp homology sequence upstream (UP) and downstream (DWN) of the region of interest is necessary for a double cross-over event and integration of the mutagenized DNA in the receiver strain. Both plasmids and gDNA can be used as donors in natural transformation. Genes coding for antibiotic resistance are in-between FRT sites, by conjugation with plasmid pFlp2 [7] expressing a Flippase we can excise the antibiotic resistance gene from *V. cholerae* strains by recombination between FRT sites. Engineered strains were confirmed by colony PCR.

**AGV4**: constructed by natural transformation. Donor plasmid pAG1, receiver strain EPV50. Strain confirmed by PCR with primers 537+538 for integration of P*_lac_::ftsZ-RFPT*, ZeoR at *lacZ* locus.

**AHV42**: constructed by natural transformation. Donor plasmid pAH3, receiver strain EPV50. Strain confirmed by PCR with primers 2162+2163 for integration of P*_seqA_::seqA-YGFP*, ZeoR at *seqA* locus.

**EGV9**: constructed by natural transformation. Donor plasmid pEG223, receiver strain EPV50. Strain confirmed by PCR with primers 537+538 for integration of P*_lac_::YGFP-ftsI*, ZeoR at *lacZ* locus.

**EGV324**: constructed using strains containing Tet homologies inserted near *ter1* and *oriC1* [6], *lacO* arrays-KanR and *parS^pMT1^*-CmR sites were added in between Tet homologies by natural transformation with plasmid pAD20 [6] and pAD39 [6], respectively. Natural transformation with pEP70 [2] was used to insert P*_lac_::lacI-RFPT-YGFP-parB^pMT1^*-ZeoR at the *lacZ* locus. Strain confirmed by PCR with primers 1514+1794 for integration of *lacO* array-KanR at *ter1* locus, primers 1655+1656 for integration of *parS^pMT1^*-CmR at *oriC1* locus, and 537+538 for integration of P*_lac_::lacI-RFPT-YGFP-parB^pMT1^*, ZeoR at *lacZ* locus.

**EGV326**: constructed by natural transformation. Donor gDNA EGV88 [2], receiver strain EGV324. Strain confirmed by PCR with primers 1857+1862 for deletion of *matP*.

**EGV346**: constructed using strains containing Tet homologies inserted near *oriC1* and *oriC2* [6], *lacO* arrays-KanR and *parS^pMT1^*-CmR sites were added in between Tet homologies by natural transformation with plasmid pAD20 [6] and pAD39 [6], respectively. Natural transformation with pEP70 [2] was used to insert P*_lac_::lacI-RFPT-YGFP-parB^pMT1^*-ZeoR at the *lacZ* locus. Strain confirmed by PCR with primers 1740+1741 for integration of *lacO* array-KanR at *oriC2* locus, primers 1655+1656 for integration of *parS^pMT1^*-CmR at *oriC1* locus, and 537+538 for integration of P*_lac_:: lacI-RFPT-YGFP-parB^pMT1^*, ZeoR at *lacZ* locus.

**EGV616**: constructed by natural transformation. Donor plasmid pEG493 for integration of P*_zn_*::*dsbA*_ss_*-mCherry*, ZeoR at *lacZ* locus and pEG500 for integration of P*_lac_::YGFP*, CmR at *hapR* locus, receiver strain EPV50. Strain confirmed by PCR with primers 537+538 for integration of P*_zn_*::*dsbA*_ss_*-mCherry*, ZeoR at *lacZ* locus, and primers 1948+1949 for integration of P*_lac_::YGFP*, CmR at *hapR* locus.

**EGV623**: constructed by integration/excision of plasmid pEG504 into receiver strain EPV50. Plasmid integration was selected by AmpR and excision by growth on LB plates + 15% sucrose and AmpS. Strain confirmed by PCR with primers 4296+4299 for integration of P*_PBP1a_::PBP1a-sfGFP* at *PBP1a* locus.

**References**

1. Galli E, Poidevin M, Le Bars R, Desfontaines J-M, Muresan L, Paly E, et al. Cell division licensing in the multi-chromosomal Vibrio cholerae bacterium. Nat Microbiol. 2016;1: 16094. doi:10.1038/nmicrobiol.2016.94

2. Demarre G, Galli E, Muresan L, Paly E, David A, Possoz C, et al. Differential Management of the Replication Terminus Regions of the Two Vibrio cholerae Chromosomes during Cell Division. PLoS Genet. 2014;10: e1004557. doi:10.1371/journal.pgen.1004557

3. van Raaphorst R, Kjos M, Veening J-W. Chromosome segregation drives division site selection in Streptococcus pneumoniae. Proc Natl Acad Sci U S A. 2017;114: E5959–E5968. doi:10.1073/pnas.1620608114

4. Altinoglu I, Merrifield CJ, Yamaichi Y. Single molecule super-resolution imaging of bacterial cell pole proteins with high-throughput quantitative analysis pipeline. Sci Rep. 2019;9: 6680. doi:10.1038/s41598-019-43051-7

5. Galli E, Paly E, Barre F-X. Late assembly of the Vibrio cholerae cell division machinery postpones septation to the last 10% of the cell cycle. Sci Rep. 2017;7: 44505. doi:10.1038/srep44505

6. David A, Demarre G, Muresan L, Paly E, Barre F-X, Possoz C. The two Cis-acting sites, parS1 and oriC1, contribute to the longitudinal organisation of Vibrio cholerae chromosome I. PLoS Genet. 2014;10: e1004448. doi:10.1371/journal.pgen.1004448

7. Galli E, Midonet C, Paly E, Barre F-X. Fast growth conditions uncouple the final stages of chromosome segregation and cell division in Escherichia coli. PLoS Genet. 2017;13: e1006702. doi:10.1371/journal.pgen.1006702
