# Supplementary material for: Recovery of Vibrio cholerae polarized cellular organization after exit from a non-proliferating spheroplast state
Source: PLoS One. 2023 Oct 26;18(10):e0293276. doi: 10.1371/journal.pone.0293276 (PMC10602287; doi:10.1371/journal.pone.0293276)
Supplement: S1 Table — (DOCX) [file pone.0293276.s033.docx]

**Supplementary Table 1**. List of bacterial strains, plasmids, linkers and primers

| **Strains** |  |  |
| --- | --- | --- |
| **Name** | **Relevant genotype or features** | **Reference** |
| AGV4 | N16961 *ChapR* Δ*lacZ::*(P*_lac_::ftsZ-RFPT-Sh ble*) zeo^R^ gm^R^ | This study |
| AHV42 | N16961 *ChapR* Δ*lacZ* P*_seqA_::seqA-YGFP-Sh ble* zeo^R^ gm^R^ | This study |
| EGV9 | N16961 *ChapR* Δ*lacZ::*(P*_lac_::YGFP-ftsI-Sh ble*) zeo^R^ gm^R^ | This study |
| EGV34 | N16961 *ChapR* Δ*lacZ* P*_ftsK_::ftsK-YGFP-Sh ble* zeo^R^ gm^R^ | [1] |
| EGV72 | N16961 *ChapR* Δ*lacZ::*(P*_lac_::YGFP-parB1-Sh ble*) zeo^R^ gm^R^ | [2] |
| EGV75 | N16961 C*hapR* Δ*lacZ* Δ*hubP* gm^R^ | [1] |
| EGV324 | N16961 C*hapR* Δ*lacZ::*(P*_lac_::lacI-RFPT-YGFP-parBpMT1*) + l*acO* array-*aph* inserted on Chr1 at position 1.52 Mb (1525700 bp) (ter1) + *parSpMT1* inserted on Chr1 at position 0.05 Mb (53355 bp) (oriC1) kan^R^ gm^R^ | This study |
| EGV326 | N16961 C*hapR* Δ*lacZ::*(P*_lac_::lacI-RFPT-YGFP-parBpMT1*) + l*acO* array-*aph* inserted on Chr1 at position 1.52 Mb (1525700 bp) (ter1) + *parSpMT1* inserted on Chr1 at position 0.05 Mb (53355 bp) (oriC1) *matP::bla* amp^R^ kan^R^ gm^R^ | This study |
| EGV346 | N16961 C*hapR* Δ*lacZ::*(P*_lac_::lacI-RFPT-YGFP-parBpMT1*) + l*acO* array-*aph* inserted on Chr2 at position 1.05 Mb (1048462 bp) (oriC2) + *parSpMT1* inserted on Chr1 at position 0.05 Mb (53355 bp) (oriC1) kan^R^ gm^R^ | This study |
| EGV616 | N16961 Δ*lacZ::*(P*_zn_*::*dsbA*_ss_*-mCherry-Sh ble*) Δ*hapR::*(P*_lac_*::*YGFP-cat*) cml^R^ zeo^R^ gm^R^ | This study |
| EGV623 | N16961 *ChapR* Δ*lacZ* P*_PBP1a_::PBP1a-sfGFP* gm^R^ | This study |
| EPV50 | N16961 *ChapR* Δ*lacZ* gm^R^ | [3] |
| EPV390 | N16961 *ChapR* Δ*lacZ* carrying the *ftsZ84* *ts* chromosomal mutation (G106S) rif^R^ gm^R^ | [1] |
| EPV453 | N16961 *ChapR* Δ*lacZ* P*_hubP_::hubP-sfGFP* gm^R^ | [2] |
|  |  |  |
| **Plasmids** |  |  |
| **Name** | **Relevant genotype or features** | **Reference** |
| pAD20 | integration-excision vector; Tet’-*lacO* array-*aph* -‘Tet; *sacB*; ori R6K; cml^R^, kan^R^ | [3] |
| pAD39 | integration-excision vector; Tet’-*parSpMT1*-FRT-*cat-FRT*-‘Tet; *sacB*; ori R6K; cml^R^, kan^R^ | [3] |
| pAG1 | P*_lac_::ftsZ-RFPT-Sh ble* flanked by the upstream and downstream regions of *lacZ*; ori pUC; amp^R^, zeo^R^ | This study |
| pAH3 | *seqA-YGFP-Sh ble* flanked by the upstream and downstream  regions of *seqA*; ori pSC101; amp^R^, zeo^R^ | This study |
| pEG223 | P*_lac_::YGFP-ftsI-Sh ble* flanked by the upstream and downstream regions of *lacZ*; ori pUC; amp^R^, zeo^R^ | This study |
| pEG493 | P*_zn_*::*dsbA*_ss_*-mCherry-Sh ble* flanked by the upstream and downstream regions of *lacZ*; ori pUC; amp^R^, zeo^R^ | This study |
| pEG500 | P*_lac_::YGFP-cat* flanked by the upstream and downstream  regions of *hapR*; ori pUC; amp^R^, cml^R^ | This study |
| pEG504 | integration-excision vector; *PBP1a-sfGFP* at native locus; *sacB*; ori R6K; amp^R^ | This study |
| pEP70 | P*_lac_::lacI-RFPT-parBpMT1-YGFP-FRT-Sh ble-FRT* flanked by the upstream and downstream regions of *lacZ*; ori pUC; amp^R^, zeo^R^ | [4] |
|  |  |  |
| **Linkers** |  |  |
| **Protein fusion** | **Linker sequence** |  |
| YGFP-FtsI | SYLE |  |
| FtsK-YGFP | LELV |  |
| FtsZ-RFPT | LELV |  |
| HubP-sfGFP | GGAAAGG |  |
| LacI-RFPT | LELV |  |
| YGFP-ParB^pMT1^ | INRTA |  |
| YGFP-ParB1 | SYLE |  |
| PBP1a-sfGFP | DLELV |  |
| SeqA-YGFP | GLELV |  |
|  |  |  |
| **Primers** |  |  |
| **Name** | **Sequence 5’-3’** | **Used for** |
| 526 | GCGGATCCTGAAAGCGTCATCCCCACAATAAGC | pEG493 |
| 537 | GCGGATCCTCGCCCACCATCGTTCGCCGCGAC | *lacZ* locus |
| 538 | ATTCCCGGGTCAACATCCGTAGGAGCATAGG | *lacZ* locus |
| 542 | GCGATGCATGGTCTGACGCTCAGTGGAAC | pAH3 |
| 1514 | TGACGAGTTCTTCTGAGCGGGACTCTGG | *lacO* array |
| 1655 | CCCAATGCATAACGGATACC | *oriC1* locus |
| 1656 | GAGGGCGGATTATAGAGAAC | *oriC1* locus |
| 1740 | CGACCATATTGCGTAGGTTC | *oriC2* locus |
| 1741 | GCGTGATCAGCAAATAGGTC | *oriC2* locus |
| 1794 | AGAATCCGCAACGTATTCCC | *ter1* locus |
| 1857 | TTCAACTAGTCGATGAAGCAAAGCTCGTTGTC | *matP* locus |
| 1862 | TTCAACTAGTAGGCGAGCTTCTTCAAGTAGCG | *matP* locus |
| 1948 | ATCCCAATGCAACTGCTCTC | *hapR* locus |
| 1949 | CAAAGTGCGTGATTGGACTC | *hapR* locus |
| 2147 | TTTTTCTCGAGGGTCCGGCTGGT | pEG493 |
| 2162 | CAATGCCGTGACGGTCGTAG | *seqA* locus |
| 2163 | CAGCAGATCGAGTCCTACAG | *seqA* locus |
| 2296 | CCCACTAGTTATTTATACAGCTCATGCATGCC | pEG500 |
| 2297 | CCTCTCGAGTTGGTGTCTAAAGGTGAAGAACTGTTCAC | pEG504 |
| 2502 | GGGCTCGAGGTCAGCTTGACGTCTCA | pAG1 |
| 2509 | TTTGCATGCATGAGCTGTATAAATCGTACCTCGAGAAGAAGAAAGCCCCAGCCAAAG | pEG223 |
| 2510 | GGGACTAGTTTATTTGTTTTGGAAGCGGTTTTCATCAGGAGC | pEG223 |
| 2895 | ATTTGCGGCCGCCTTGAGGGATCTGAAGTTCC | pAH3 |
| 2896 | CCGACAGTAAGACGGGTAAGCCTGTTGATG | pAH3 |
| 2897 | GTTCCACTGAGCGTCAGACCATGCATCGCTGCGTGACGTGGGCTTTTG | pAH3 |
| 2898 | CTTCACCTTTAGACACCAACTCGAGCCCAATGGAATGGGTAACTTTCTCAA | pAH3 |
| 2899 | GGGCTCGAGTTGGTGTCTAAAGGTGAAG | pAH3 |
| 2900 | CGGAATAGGAACTTCAGATCCAAGCTTGCTAGCTATTTATACAGCTCATG | pAH3 |
| 2901 | GCTAGCAAGCTTGGATCTGAAGTTCCTATTCCG | pAH3 |
| 2902 | GGAACTTCAGATCCCTCAAGGCGGCCGCAAATATTTTGCTGATTACAGCCCAA | pAH3 |
| 2903 | CATCAACAGGCTTACCCGTCTTACTGTCGGCCAAAAGCCAAGTCGTATTCA | pAH3 |
| 3331 | AACCCATCACATATACCTGCCGTTC | pEG504 |
| 3340 | CACATGTGGAATTCCCATGTCAGC | pEG504 |
| 3707 | CTGACATATGATGTCTAAAGGTGAAGAACTG | pEG500 |
| 4002 | TAACAAGTCATCAATAAAGCCA | pEG493 |
| 4005 | ACTCCTTATGGCTTTATTGATGACTTGTTATATTAGTTAATCAGTACTCGAGATCC | pEG493 |
| 4006 | TTATTGTGGGGATGACGCTTTCAGGATCCGCCAATGTTAGTCAGATGGACACTTAAGGC | pEG493 |
| 4229 | CAAGCTTGTCGACTTATTTGTACAGCTCATCCATGC | pEG493 |
| 4271 | GGAGGCCTACTAGTTTATTTGTAGAGTTCATCCATGCCG | pEG504 |
| 4273 | CAGGCCTACTAGTTTATTTGTACAGCTCATCCATG | pEG504 |
| 4290 | GACATCCTCGAGCTCGTGTCCAAGGGCGAGGAGGA | pEG504 |
| 4293 | TTTGCTAGCAGGAGGAATTCACCTTGTTTGAACCGATGATGG | pAG1 |
| 4296 | ATAGTGAACGGCAGGTATATGTGATGGGTTCGTGAAGTGGGTTTGGAT | pEG504 / *PBP1a* locus |
| 4297 | CTCGCCCTTGGACACGAGCTCGAGGATGTCGAACAGCTCTTCACCAGAG | pEG504 |
| 4298 | GCTGTACAAATAAACTAGTAGGCCTGTAAACTCTGGTACAGAAAACCG | pEG504 |
| 4299 | TTAACGGCTGACATGGGAATTCCACATGTGAACGACTTTGCTTGAGCGC | pEG504 / *PBP1a* locus |

**References**

1. Galli E, Poidevin M, Le Bars R, Desfontaines J-M, Muresan L, Paly E, et al. Cell division licensing in the multi-chromosomal Vibrio cholerae bacterium. Nat Microbiol. 2016;1: 16094.

2. Galli E, Paly E, Barre F-X. Late assembly of the Vibrio cholerae cell division machinery postpones septation to the last 10% of the cell cycle. Sci Rep. 2017;7: 44505. doi:10.1038/srep44505

3. David A, Demarre G, Muresan L, Paly E, Barre F-X, Possoz C. The two Cis-acting sites, parS1 and oriC1, contribute to the longitudinal organisation of Vibrio cholerae chromosome I. PLoS Genet. 2014;10: e1004448. doi:10.1371/journal.pgen.1004448

4. Demarre G, Galli E, Muresan L, Paly E, David A, Possoz C, et al. Differential Management of the Replication Terminus Regions of the Two Vibrio cholerae Chromosomes during Cell Division. PLoS Genet. 2014;10: e1004557. doi:10.1371/journal.pgen.1004557
